# What is groundwater?

**DOI:** 10.1007/s10040-026-03056-9

**Published:** 2026-04-01

**Authors:** Mark O. Cuthbert

**Affiliations:** School of Earth and Environmental Sciences, Cardiff University, Cardiff, UK

**Keywords:** Socio-hydrogeology, History of hydrogeology, Socio-economic aspects, Sustainability, Evolution of modern groundwater

## Abstract

Groundwater may be the most important substance on Earth that most people have never heard of, yet it has quietly influenced the evolution of human culture since its earliest beginnings. The predominant physicalist ontology of modernity has created such a powerful conceptual representation of groundwater, that seemingly incredible benefits have accrued to society globally via our ability to understand and relate to it in this mode. However, this modern ontological lens goes unnoticed and unannounced in most groundwater research. This study aims to broaden existing narratives about what groundwater was, is and could be, by exploring how groundwater has come to be perceived, understood and defined by WEIRD (Western, educated, industrialised, rich, democratic) societies in which the author is embedded. By considering the co-evolution of four fields of human-groundwater development, namely - behaviour, systems, psychology, and culture - this paper explores how originally participatory ways of perceiving and knowing groundwater gave way to increasingly abstracted propositional modes of knowledge. Eventually many humans came to believe that the abstract symbols and modelled quantities which they used to represent reality were actually reality itself. Thus ‘modern groundwater’ is now commonly defined and related to as a resource, a ‘thing’ devoid of inherent meaning or value, separate from ourselves, which can be manipulated to our own ends. Greater epistemic reflectivity, and a pivot to working metaphysically with groundwater as a relational process, may lead to more effective responses to some of the global predicaments we currently face.

**Supplementary Information:**

The online version contains supplementary material available at https://doi.org/10.1007/s10040-026-03056-9.

## RESUME

L’eau souterraine est peut-être le bien le plus important sur Terre dont la plupart des gens n’ont jamais entendu parler, et pourtant, elle a discrètement influencé l’évolution de la culture humaine depuis ses origines. L’ontologie physicaliste prédominante de la modernité a engendré une représentation conceptuelle si puissante de l’eau souterraine que la société mondiale a bénéficié d’avantages considérables grâce à notre capacité à la comprendre et à interagir avec elle de cette manière. Or, cette perspective ontologique moderne passe inaperçue et n’est pas mentionnée dans la plupart des recherches sur l’eau souterraine. Cette étude vise à enrichir les récits existants sur ce que l’eau souterraine a été, est et pourrait être, en explorant comment elle a été perçue, comprise et définie par les sociétés « OEIRD » (occidentales, éduquées, industrialisées, riches et démocratiques) au sein desquelles l’auteur évolue. En considérant la coévolution de quatre domaines liés à l'exploitation des eaux souterraines par l'homme – le comportement, les systèmes, la psychologie et la culture –, cet article explore comment les modes participatifs de perception et de connaissance des eaux souterraines ont initialement cédé la place à des modes de connaissance propositionnels de plus en plus abstraits. Nombreux sont ceux qui ont fini par croire que les symboles abstraits et les quantités modélisées qu'ils utilisaient pour représenter la réalité constituaient la réalité elle-même. Ainsi, les « eaux souterraines modernes » sont aujourd'hui communément définies et perçues comme une ressource, une « chose » dépourvue de sens ou de valeur intrinsèque, distincte de nous-mêmes, et que nous pouvons manipuler à nos propres fins. Une plus grande réflexivité épistémique et une approche métaphysique des eaux souterraines, envisagées comme un processus relationnel, pourraient permettre d'apporter des réponses plus efficaces à certains des problèmes mondiaux auxquels nous sommes confrontés.

## RESUMEN

El agua subterránea puede ser la sustancia más importante en la Tierra que la mayoría de la gente nunca ha oído mencionar, que ha influenciado silenciosamente en la evolución de la cultura humana desde sus inicios. La ontología fisicalista predominante de la modernidad ha creado una representación conceptual tan poderosa de las aguas subterráneas, que beneficios aparentemente increíbles han llegado a la sociedad a nivel global gracias a nuestra capacidad para entenderla y relacionarnos con ella en este modo. Sin embargo, esta perspectiva ontológica moderna pasa desapercibida y no anunciada en la mayoría de las investigaciones sobre aguas subterráneas. Este estudio pretende ampliar las narrativas existentes sobre qué era, es y podría ser el agua subterránea, explorando cómo el agua subterránea ha llegado a ser percibida, entendida y definida por las sociedades WEIRD (occidentales, educadas, industrializadas, ricas, democráticas) en las que el autor está inmerso. Al considerar la coevolución de cuatro campos del desarrollo humano-agua subterránea, a saber: comportamiento, sistemas, psicología y cultura, este artículo explora cómo las formas originalmente participativas de percibir y conocer el agua subterránea dieron paso a modos proposicionales de conocimiento cada vez más abstractos. Con el tiempo, muchos humanos llegaron a creer que los símbolos abstractos y las cantidades modeladas que usaban para representar la realidad eran en realidad la realidad misma. Así, el 'agua subterránea moderna' se define y se relaciona comúnmente como un recurso, una 'cosa' carente de significado o valor inherente, separada de nosotros mismos, que puede ser manipulada para nuestros propios fines. Una mayor reflectividad epistémica y un giro hacia trabajar metafísicamente con las aguas subterráneas como un proceso relacional pueden conducir a respuestas más efectivas a algunos de los problemas globales que enfrentamos actualmente.

## Abstract (zh)

地下水可能是地球上最为重要、却又为大多数人所忽视的一种物质。然而, 自人类文明诞生之初, 地下水便在不知不觉中深刻影响了人类文化的演进。现代性所主导的物理主义本体论构建了一种极具影响力的地下水概念框架, 使人类能够以此方式理解和利用地下水, 并由此在全球范围内获得了看似不可思议的社会效益。然而, 这种现代本体论视角在大多数地下水研究中往往是隐含的、未经反思的。本文旨在拓展关于“地下水是什么、曾经是什么以及可能成为什么”的既有叙事, 通过考察作者所处的 WEIRD 社会 (即西方、受过良好教育、工业化、富裕且民主的社会) 中, 人们如何感知、理解和界定地下水, 来反思地下水认知方式的形成过程。通过分析人类—地下水关系在行为、系统、心理和文化这四个领域中的协同演化, 本文探讨了人类最初以参与式方式感知和认识地下水的路径, 如何逐渐被日益抽象化的命题式知识体系所取代。最终, 许多人开始将用于表征现实的抽象符号和模型化量值误认为现实本身。因此, “现代地下水”通常被界定和对待为一种资源、一种缺乏内在意义和价值的“客体”, 被视为与人类自身相分离、可供操控以服务于人类目的的存在。通过增强认识论反思, 并转向从形而上学层面将地下水视为一种关系性过程来加以理解, 或可为应对当前人类所面临的一些全球性困境提供更为有效的路径。

## RESUMO

A água subterrânea pode ser a substância mais importante da Terra da qual a maioria das pessoas nunca ouviu falar, embora tenha influenciado silenciosamente a evolução da cultura humana desde seus primórdios. A ontologia fisicalista predominante na modernidade criou uma representação conceitual tão poderosa da água subterrânea que benefícios aparentemente incríveis foram acumulados pela sociedade globalmente por meio de nossa capacidade de compreendê-la e nos relacionarmos com ela dessa maneira. No entanto, essa lente ontológica moderna passa despercebida e não é mencionada na maioria das pesquisas sobre água subterrânea. Este estudo visa ampliar as narrativas existentes sobre o que a água subterrânea era, é e poderia ser, explorando como ela passou a ser percebida, compreendida e definida pelas sociedades WEIRD (ocidentais, educadas, industrializadas, ricas e democráticas) nas quais o autor está inserido. Ao considerar a coevolução de quatro campos do desenvolvimento humano-água subterrânea, a saber – comportamento, sistemas, psicologia e cultura – este artigo explora como as formas originalmente participativas de perceber e conhecer a água subterrânea deram lugar a modos de conhecimento proposicionais cada vez mais abstratos. Com o tempo, muitos seres humanos passaram a acreditar que os símbolos abstratos e as quantidades modeladas que utilizavam para representar a realidade eram, na verdade, a própria realidade. Assim, a "água subterrânea moderna" é hoje comumente definida e relacionada como um recurso, uma "coisa" desprovida de significado ou valor inerente, separada de nós, que pode ser manipulada para nossos próprios fins. Uma maior reflexão epistêmica e uma mudança para o trabalho metafísico com a água subterrânea como um processo relacional podem levar a respostas mais eficazes para alguns dos dilemas globais que enfrentamos atualmente.

## Introduction

Groundwater is a concept that few people fully appreciate, yet has played a central, if hidden, role in the history of life on this planet, and on the emergence of human culture as we know it. The deepest primordial groundwater lubricates the tectonic plates which provide the mosaic on which life arose (Shillington 2018), and the circulation of saline groundwater in black smokers provided the most likely candidate for life to have first evolved on Earth (Martin et al. 2008). Several million years ago, groundwater fed springs in the East African Rift System enabled survival and competition of early hominins driving early hominin speciation (Cuthbert and Ashley 2014, Cuthbert et al. 2017), and later mediated their global dispersal in the Paleolithic (Faure et al. 2002). Groundwater myths and water beings were thought to be a central part of the emerging cultural codes that were key to survival and thriving through the early Neolithic (Strang 2023). The agricultural revolution which set the stage for the axial revolution and the rise of early civilisation was enabled by groundwater by providing water in otherwise dry places and the birth of the nation state by supporting sufficient densities of people along key trade routes (Issar 2008, Angelakis et al. 2016). Now groundwater supports half of the world’s irrigated agriculture, half the world’s drinking water, is integral to the rise of megacities and global commerce and industry in both the carbon and post-carbon economy (UNESCO 2022). And all the time this profound hidden substance that shapes us remains out of sight and all too often out of mind; but creeping, seeping and moulding us in a reciprocal way nevertheless.

Reach for a hydrogeology text book and you’ll find a definition of groundwater along the lines of *“subsurface water that occurs beneath the water table in soils and geologic formations that are fully saturated” (*Freeze and Cherry 1979*)* Furthermore, common definitions of what an aquifer is often refer to “*economically useable quantities of groundwater*” (Van der Gun 2022). Thomas Kuhn argued that textbooks reveal a scientific community’s working paradigm (Kuhn 2012); in this case, such a conceptualisation of groundwater stems from the ‘modern’ cultural paradigm of philosophical materialism born in the scientific revolution and the Enlightenment of 17^th^ century Europe. Note that in this paper the word ‘modern’ is not a reference to the residence time of recent (e.g. ‘post-bomb’) age groundwater (Gleeson et al. 2016), but rather to the cultural stage or historic period of modernity. The data show that while modernity has been characterised by sustained increases in some, mostly exterior, metrics of human flourishing (Pinker 2018), this has been achieved directly at the expense of causing environmental and societal ‘crises’ which now pose existential threats to multiple Earth systems (Raworth 2012). From a groundwater perspective, the widespread impact of over-pumping on ecology, land subsidence, salinization, and deterioration in water quality are now well documented (Gleeson et al. 2020), although the distribution of such impacts is uneven and renewable groundwater use is also under-utilised in many parts of the world (Jude 2020, Cuthbert et al. 2023, 2024). A salient recent discovery is that, primarily driven by food production via irrigation enabled by fossil fuel use, we have now moved so much groundwater from the continents to the oceans that we have altered the tilt of the Earth’s rotation (Seo et al. 2023). Our impact on groundwater is occurring on a truly planetary scale.

The so-called ‘meta-crisis’ – that complex, interrelated set of global ecological, economic, political, moral, and existential crises - characterises much commentary on our current global situation (Ramos 2011, Hedlund 2023, Rowson 2023). Rowson (2023) sensibly questions the wisdom of letting such an anti-framing dominate our responses to the predicaments of the Anthropocene. However, the meta-crisis rhetoric does useful work if it causes us to pause for thought to reflect and critique the cultural mode and moment that hydrogeology has occupied since its emergence over the past 200 years - one that is predominantly immersed in modernity. This paper aims to provoke consideration of whether our whole way of thinking needs an upgrade, and that simply improving our hydrogeological knowledge and skills is insufficient for the challenges we face, even if we now do so in an increasingly more nuanced ‘systems thinking’ way. It may be a useful analogy to suggest we need to upgrade our operating system, and not just re-write the code we’re running in ever elaborate ways, while caveating that using machine metaphors for the process of being human is perhaps more than a little dangerous, as will become evident later in the paper.

As a species, our ability to “*know ourselves*” (Murray et al. 2017) as “*self-interpreting animals*” (Taylor 1985), is perhaps our most distinguishing feature – in essence it is our ability to form and work with conceptual ideas that has enabled us to thrive in a complexifying, global, world-system. We have evolved to inhabit thought-worlds (a.k.a. worldviews, cultural paradigms, myths, consensus realities, social imaginaries); lenses through which we sense, perceive and interpret our reality and which determine our values and goals. Explicitly recognising and naming the dominant thought-world (or worlds) we take for granted is surely critical for, as Whitehead said, “*As we think, we live*” (Whitehead 1938) i.e. what we think groundwater *is* determines how we experience and interact with it.

**Fig. 1:**
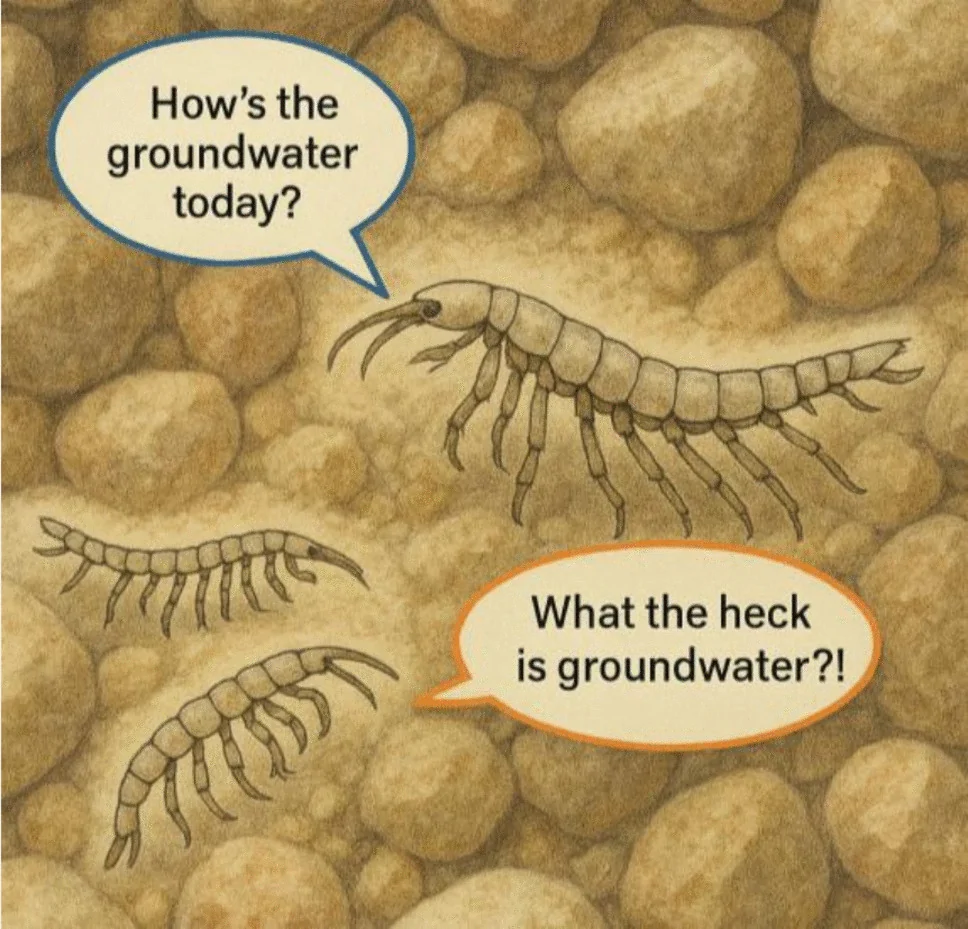
Groundwater (stygo)fauna are metaphorically (as well as very often physically) ‘blind’ to groundwater. Many myths and worldviews live through us implicitly without meta-conscious reflection. What are we blind to and how does that impact our view of groundwater?

Hence, as Fig. 1 illustrates, to a greater or lesser extent, there is a challenge to truly seeing what is around us since we are often blind to the worldview in which we are immersed as it is the very air we breathe or, rather, the (ground)water we swim in.

This paper seeks to sketch an alternative narrative for understanding the evolution of ‘modern groundwater’ in the context of evolution more broadly, including the evolution of culture and consciousness (please see the electronic supplementary material (ESM) for a definition of how this and other key terms are used in this paper to avoid potential misunderstandings). This study is an encouragement towards new *ways* of thinking that are consistent with the trajectory of evolution in its broadest sense, and which may enable us to deliberately transcend and include previous stages of behavioural, systemic, psychological and cultural developments. Despite framing the title ‘What *is* groundwater?’, my purpose is not to make a case for any particular meaning of groundwater. Rather, it is to encourage us to reflect on our own, often unacknowledged, epistemological (i.e. theories of knowing) and ontological (i.e. theories of being) assumptions, what that might mean for our own relationship to groundwater, and how as a community we can open to new possibilities. That said, in the latter part of the paper, a way of seeing the world is also briefly proposed which has potential to out-compete the physicalist metaphysics which dominates much modern academic discourse.

From an evolutionary standpoint, global sustainability in the Anthropocene has scarcely been explored (Søgaard Jørgensen et al. 2024), and no literature (that the author is aware of, (Zhang et al. 2024) notwhithstanding) takes up the metamodern challenge to integrate such ideas with emerging theories on ‘stages of development’ or a focus on ‘interiors’ (see later definitions). Disciplinary barriers (Ellis 2015) and cultural inertia in the current paradigm of normal-science (Kuhn 2012) are formidable challenges, so while the meta-modern stance outlined in the paper is likely heretical with respect to both the modern and post-modern paradigms that dominate academia, it seems important to attempt to breach them. It is an attempt to meet the challenge that we need new ideas in our field and not just more of the same (Editor 2021), to move beyond ‘zombie-science’ (Schwartz 2013), and to reveal more explicitly our foundational assumptions (Venot et al. 2022). It is a deliberate attempt to push the boundaries of what socio-hydrogeology can be. It is perhaps an over-ambitious aim with the potential to scandalise some specialists across the sometimes disparate fields that are touched upon in attempting a paper with this breadth. So, while it may open up as many questions as it answers, it is hoped that it will provoke some useful discussions and ways forward for this precious and mysterious phenomena we call groundwater.

Responding to the challenge by Ananda & Storm (Storm 2021) that “*scholars should work to become conscious of their own values to make their motivations explicit*” I now declare my own ontological bias as living deliberately within a monist, consciousness-first metaphysics (Henry 2005, Kastrup 2018), and a rejection of mainstream physicalism. Although this bias will become apparent in the discussion later, for the bulk of the paper, I attempt to put that particular worldview to one side and write within an emerging paradigm of metamodernist thought (see methods) to which I am sympathetic but not committed to, beyond exploring what useful work it can be put to for this study. The motivation for writing this will be, rightly, questioned by some. So, from the outset I want to iterate that I am only intending to speak into the origins of the culture of which I am a part, into which I was enculturated, and with which I now carry an ability to critique from within. Any inferences I make based on insights from the study of other extant cultures is not an attempt to appropriate, though the power at play in my wielding my own position as a WEIRD (western, educated, industrialised, rich, democratic) (Henrich et al. 2010a***, ***b) academic (with English-Celtic-Nordic ancestry) to publish this paper is evident. When I use the word ‘we’ I do not assume that I can speak for you. But I hope that, by using such language in some places in the paper, any readers who also feel aligned as an inheritor of WEIRD culture may feel a sense of solidarity by that turn of phrase and find useful challenge in what I am saying as a result.

The methods section outlines the approach that has been taken to the problem in general terms to develop and justify a hermeneutic which is then applied to the question ‘What is groundwater?’ as presented in the Results section. The paper finishes by then framing some implications for groundwater research and the emerging field of socio-hydrogeology.

## Methods

### Overview of a metamodern hermeneutic

The challenges and possible incoherence in attempting to construct any kind of grand historical narrative are substantial (Graeber and Wengrow 2021, Storm 2021). However, any study of groundwater will necessarily be set within in an evolving socio-cultural-economic landscape in time and space and, to the extent it is even thought about at all, the landscape can seem bewilderingly complex and uncertain. So, to help us navigate, a series of methods of interpretation have been chosen to provide a map to guide the analysis. As with any map, these hermeneutic tools are designed to orientate and simplify the landscape for a certain purpose – in this case, to investigate the question ‘What is groundwater?’. Hence, the resolution and scale of the map is relative to this aim and the vantage point we are trying to achieve (Severan 2021). Being explicit about this helps to avoid ‘confusing the map with the territory’ it is attempting to describe (Korzybski 1933), by which it is meant coming to believe that the abstract symbols with which we represent reality are actually reality itself, an inclination that modernity has unfortunately inclined many of us to (McGilchrist 2021).

The two main keys to the map are to first delineate four fields of societal development and, second, to populate these using a multi-level evolutionary framework which sees them interdependently co-evolve in time and space. The framework is presented without reference to groundwater in the first instance and then applied to socio-hydrogeology in the following Results section. The result, it is hoped, is a more radical and nuanced attempt to understand the evolution of groundwater than has previously been attempted.

Note, this is not an attempt to explain groundwater’s evolution for all cultures for all times, but rather the specific pathway that has led to the modern view of groundwater that many practising scientists share, at least those of us who are WEIRD, but also those other (e.g. colonised) parts of the world on to whom WEIRD values have been thrust (see (Bourke 2025) and references therein), or have self-adopted a certain subset of cultural values inherent in WEIRD culture (e.g. the western scientific method, capitalist thought, or the commodification of nature). Nevertheless, it is hoped that some of the suggested framing may be of use for others seeking to understand their own cultural development trajectory or pathway. Also, note that the intention here is not to outline the details of the development of groundwater technology through history – there are already many wonderful books and papers on that topic - rather the focus is on the evolution of human consciousness as relevant to how groundwater has been experienced and conceived.

A primary hermeneutic used in the analysis is the emerging metamodernist cultural paradigm (Vermeulen and Van den Akker 2010, Freinacht 2017, Andersen 2019, Björkman 2019, Freinacht 2019, Severan 2021, Storm 2021). Metamodernism is a structure of feeling (Vermeulen and Van den Akker 2010) that declares no particular ontological commitment (‘both-neither’) but, at least in some forms (there are at least 6 - see Freinacht in (Rowson and Pascal 2021)), proposes a map of the evolution of both human interior and exterior perspectives which, perhaps for the first time in human history, enables us to take part in a meta-conscious way in our own ongoing process of evolution. The essence of metamodernity is the return of transcendence and dimensionality (Severan 2021); it seeks to transcend all previous cultural paradigms and re-build them by including and accommodating multiple views within an evolutionary framework. This exhorts us to re-enchant the cosmos, to re-member and re-value other forms of knowing that we have forgotten, to re-embrace hierarchies of growth but not of dominance/power, to recognise a mutual unfolding of interior and exterior life which reaches to the core of whatever reality is, to reach for increased epistemic depth, and to embrace the mythopoetic.

### Four fields of development

**Fig. 2:**
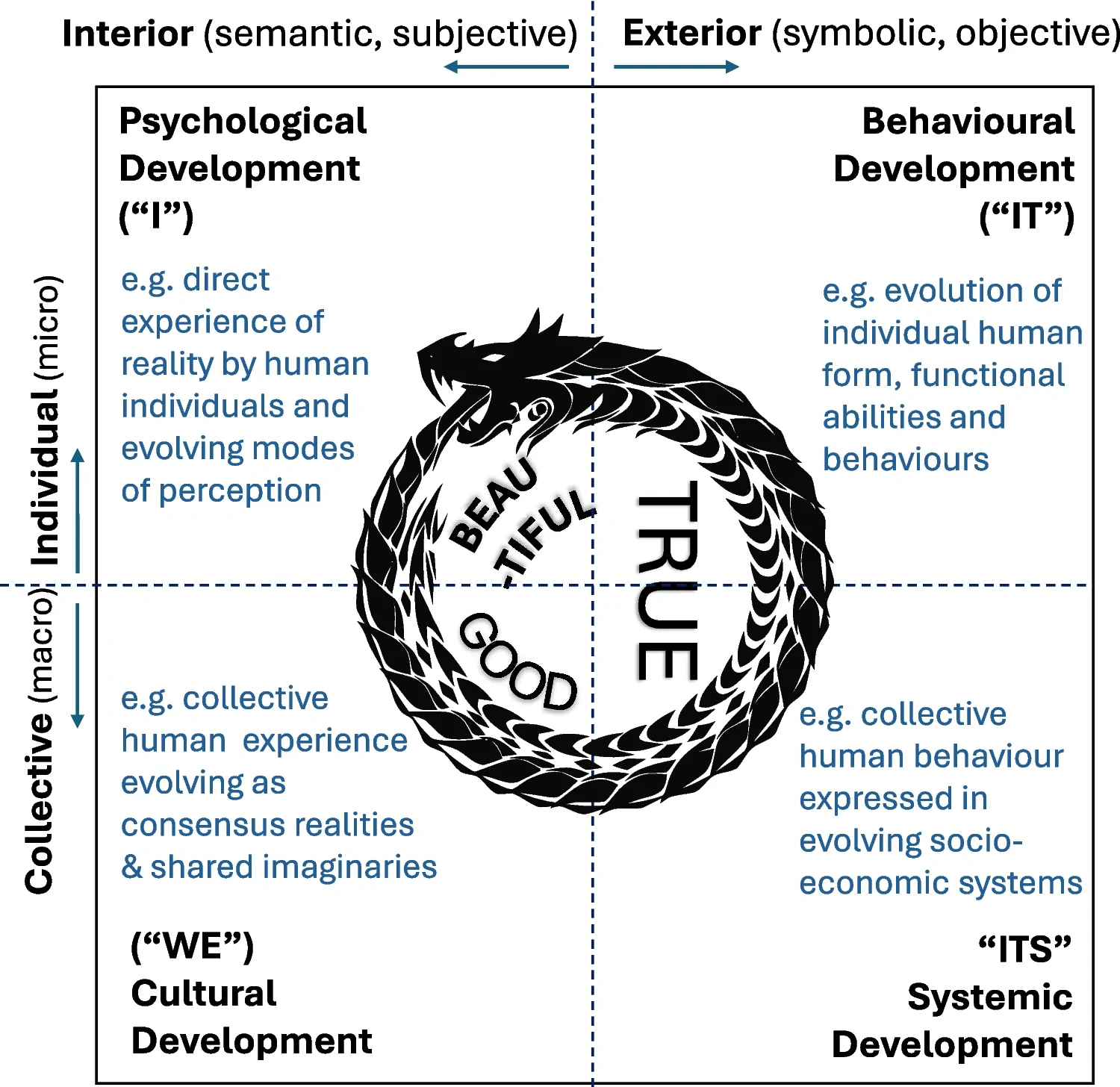
Four fields (quadrants) of development defined by combinations of interior versus exterior, and individual versus collective, aspects of enquiry. Here the quadrants have been populated specifically to focus on the development of human psychological, behavioural, cultural and systemic aspects relevant to groundwater. The correspondences to the Platonic triad of ‘the true, the beautiful and the good’ is also indicated, with an ouroborus to remind us that all four fields co-evolve and cannot ultimately be treated as separate from one another. The ouroboros image is licensed under a Creative Commons CC0 1.0 Universal License. A more detailed description of the quadrant methods are given in the ESM

To frame the development of the human relationship with groundwater, a quadrant map (Fig. 2) is first used to divide our view of reality along two axes: a vertical dimension separating the micro (small scale, individual) from the macro (large scale, multiplicity), and a horizontal dimension to separate interior (1^st^ person, experienced from the ‘inside’) and exterior (3^rd^ person, described from the ‘outside’) views. Such maps have a long history of application in social theory, though often in tri-partite form, for instance corresponding to Platonic triad (but see (Martin 2016)) of ‘the Good’ (≈lower left), ‘the True (≈right hand side) and ‘’the Beautiful’ (≈upper left). These also relate to Popper’s mental (≈upper left), physical (≈upper right), and social/systemic (≈lower quadrants) worlds each with their own criteria for validity claims (i.e. normative (‘good’), propositional (‘true’) and subjective (‘beautiful’) truths respectively). Note, however, the correspondence between such maps is not exact, since they are constructed for different sorts of perspective-taking or navigation.

To investigate the relationships between different dimensions of human-groundwater development the quadrants are delineated here as four ‘fields’ of behavioural, systemic, psychological and cultural development, following Freinacht (2019). These fields/quadrants, defined in Fig. 2, are intrinsically interrelated and interdependent. For mapping purposes this delineation serves the purpose of exploring the co-evolving nature of the human relationship with groundwater, while noting that privileging or ignoring a single field of development will lead to a ‘flattened’ view of reality (i.e. a loss of dimensionality).

Since the upper left quadrant representing the inner world of individuals has largely been neglected to date in academic scholarship on sustainability (Ives et al. 2020) despite lying at the heart of any motivation regarding actions that change our relationship with the environment (Ankrah et al. 2023)), it has been given a strong focus in this paper. How can we have “*A good life for all within planetary boundaries*” (O’Neill et al. 2018) if we don’t integrate knowledge of the evolution of our inner life into our thinking about sustainability? Internal, felt responses to external factors within the life of an organism are completely fundamental to what we might mean by well-being, and two people (for example) might have completely different psychological responses to the same external conditions.

### Evolution in time and space

Evolutionary theory is then applied within each of the four fields of development to consider how behavioural, systemic, psychological and cultural development co-emerge in time and space. This takes us into the territory of ‘stage theories’ for human and cultural development which is full of potential misunderstandings and misuses if not properly understood with openness, humility and sufficient nuance (see the ESM for more details).

The approach taken here is predicated on the idea that something akin to multi-level natural selection (Waring et al. 2024, Wilson 2020, Boyd and Richerson 2024) occurs in all quadrants simultaneously and interdependently. For instance, cultural change can be understood as an evolutionary process of variation, selection and inheritance with a ‘cultural stage’ being analogous to the concept of a species in biology, along with the associated difficulties in defining the term in a completely generic sense. As with scholars of biological human evolution there are ‘lumpers’ who shy away from making too precise a categorisation based on limited fossil evidence, and the ‘splitters’ who are happy to defend more detailed delineation of species (Stringer 2011, Dunbar 2014). My predilection is for lumping, so a 3 stage sequence is proposed although each stage has also been divided more tentatively into sub-stages for the sake of readers curious to see how creating such substages might be approached, in a way that is consistent with previous literature on stage development of culture (Wilber 1995, Freinacht 2017). This attempts to integrate usefully the key developments in human relationships with groundwater and provides a tractable map for initial explorations of the territory. With the biological concept of species, there are no hard boundaries across a small number of generations – it is only with sufficient genetic ‘distance’ (via long periods of elapsed time) that we might pronounce a speciation to have occurred. So too, in this way of viewing cultural evolution – clearly there is a continuum, and indefinite combinations of interim hybrid mixtures of cultural expressions, including the rise and fall of many counter-cultural movements in any given stage. Ultimately though, it is only with sufficient ‘distance’ that we can determine something substantially different has emerged.

Working with metamodern developmental stage theory rather than ‘ages’ (i.e. specific time historical periods) is powerful since it is implicit that communities operating at different stages will co-exist and relate to each other, which thus provides a lens through which to understand water conflict and cooperation at any spatio-temporal scale. It also gives the flexibility to consider multiple evolving ‘lines’ within each quadrant (i.e. specific aspects of development), and their interrelationships, to avoid the need to over-reach on a given metanarrative. Despite there being much existing scholarship in each quadrant and for each stage, there appears to be little peer-reviewed research which weaves these aspects together with respect to water (Hamiche et al. 2016) and no research the author is aware of which does this for groundwater specifically. And yet, this is a potentially powerful hermeneutic tool that the emerging field of socio-hydrogeology could draw on. Here the intention is to consider the branch of consciousness that hydrogeology is predominantly flowing in, not to argue for a generic model of cultural evolution that applies across the board. The proposal of groundwater evolving within a progression of stages that sit in a growth hierarchy is not intended to imply that other paths of growth were not possible (or not lost to us due to lack of evidence), or that this trajectory was inevitable.

A summary of the key scholarship on which the study draws, focussed on the main lines of evolutionary theory of interest to the present work within each quadrant, is given in the ESM .

## Results

### Flawed notions of modern groundwater’s evolution

Before trying to provide an alternative, some reflection is warranted on the flaws of the predominant narrative about modern groundwater’s evolution. It is not setting up a straw man to propose that the story of groundwater is most often told in the same way that the story of human interaction with nature more broadly is often told. (Loftin and Leyf 2023) put it well [my additions]:

*“the story is most often as follows: ancient humans lived in ignorance of the actual causes of natural phenomena [such as groundwater], and, being ruled by fear and superstition, projected the dynamics of their own psyches onto the outer world, anthropomorphising all things and contriving to see will and agency behind everything that happened. Eons of superstitious animism elapsed before, almost of a sudden, people were delivered from their beliefs by the advent of the scientific method…Therefore as science expanded human knowledge about nature, human perception of nature has been supposedly freed from the conditioning influence of false beliefs, resulting in material objects moving in conformity to natural laws. …This narrative, or one much like it has …found its way to a seemingly permanent place in the Western imaginary through the work of popularizers of science and history.” (*Loftin and Leyf 2023*)*

So, while existing histories of hydrogeology give nice summaries of what ancient people did, or wrote about, with respect to using groundwater in the past, the focus is almost always about the evolution of the *contents*, and almost never on the *mode*, of consciousness. There is also now an unfortunate back-catalogue of “serious misinformation” of ancient experience and thought regarding groundwater which has been recently and comprehensively articulated/corrected (Koutsoyiannis and Mamassis 2021). Hence, how our ancestors experienced groundwater, and what they thought about groundwater, is largely obfuscated by the most easily available and widely disseminated hydrological literature. Our own modern mode of ‘scientific’ thinking goes unannounced and forms a presumptive backdrop not just to our work in the present but also unquestioningly projected back into the past (Fetter 2004a, b). This also happens knowingly, and with fundamentalist evangelical zeal, by those academics who promulgate the view of science as “how we know what’s really true” while belittling the mythic world of more ancient minds as having little to say about truth or reality (e.g. Dawkins 2012).

This habit of projecting modern consciousness onto ancient minds, termed “logomorphism” (Barfield 1973, 1988), was widespread in much 20^th^ century anthropology which is perhaps responsible for transference across to the 20^th^ century hydrogeological literature. While more recent anthropological scholarship has moved on from such notions, this still looms large in the popular imagination. In essence, common hydrogeological textbook notions are driven from the right hand quadrants of behavioural and systemic development, privileging an unacknowledged ‘scientism’ born in logical positivism and substance physicalism (materialism). Psychological and cultural development from a 1^st^ person perspective are rarely considered, and cultural aspects are almost only ever considered from a 3^rd^ person perspective. While laudable historical overviews (e.g. (Gleick 2023)) are full of useful information about the evolution of water technology, they only ever deal with exterior right-hand quadrants.

**Fig. 3:**
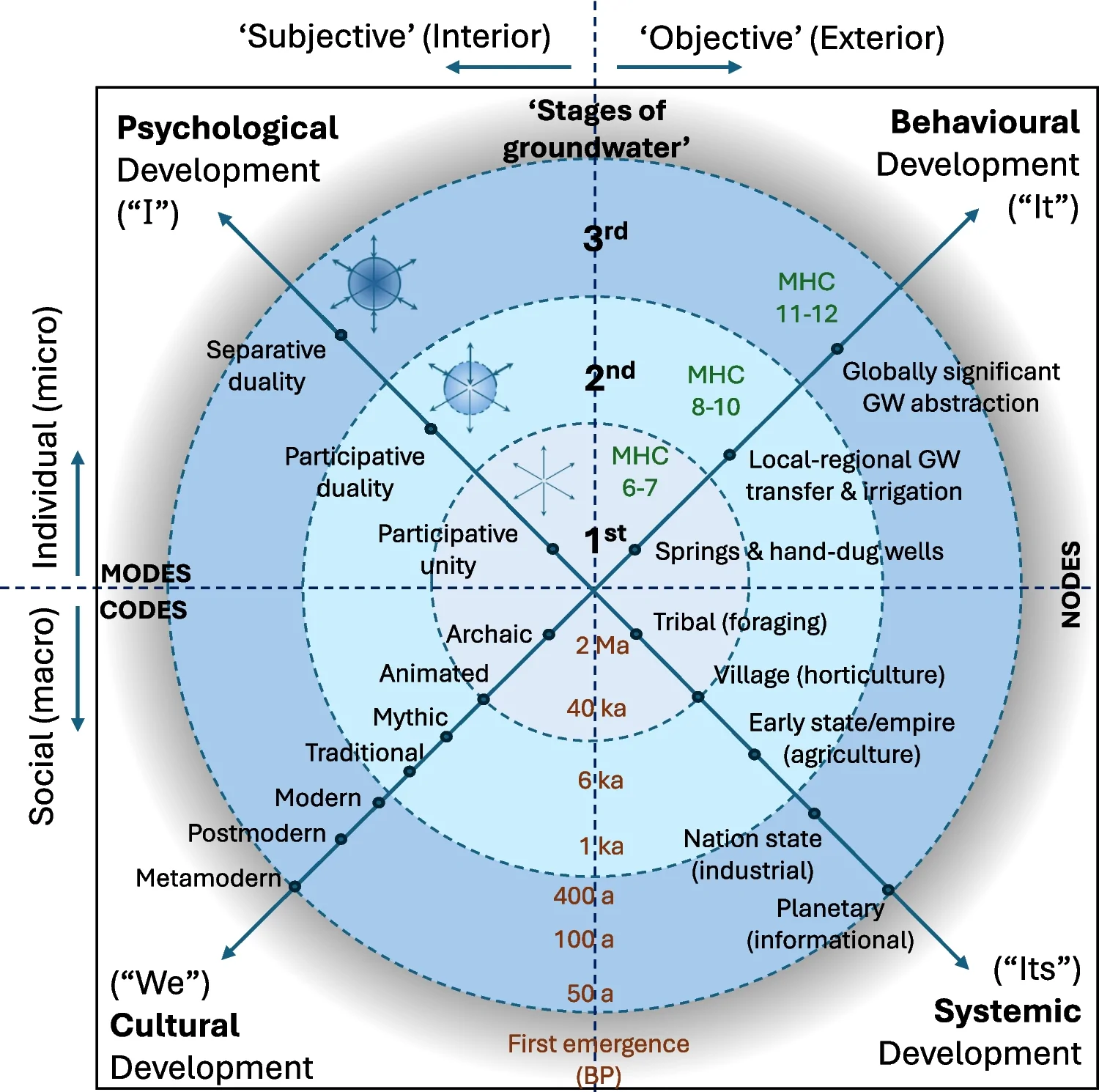
Four fields and three major stages of co-evolving human-groundwater development involved in the appearance of modern groundwater. Timelines are approximate first developments in consensus reality, boundaries are fuzzy and nominal. Right hand quadrants represent developing complexity in interdependence between objective exterior ‘nodes’ which are experienced via upper left quadrant ‘modes’ of consciousness filtered and modified within lower left quadrant cultural ‘codes’. MHC = model of hierarchical complexity (Commons, Trudeau et al. 1998). Lower right quadrant synthesizes stages from (Wilber 1995, Ellis 2015, Freinacht 2017, Waring et al. 2024). Lower left synthesises stages from (Wilber 2000, Freinacht 2017)

A major reason for this is appears to be that science is so often associated with a naïve physicalist (materialist) metaphysics that the two have become enmeshed not just in the public imagination, but even for many working scientists, including groundwater scientists. While the definition of ‘science’ is not straightforward (Ladyman 2012), by this author it is being used here to mean the systematic study of the behaviour of perceived phenomena, with a view to being able to predict other perceived phenomena (consistent also with Einstein’s sensibility). In this sense, science itself makes no metaphysical claims about what (we call) nature *is*, but rather about how nature *behaves* (Kastrup 2021a, b). In the language of the quadrants (developed in the Methods section above), the necessary epistemological stance we must all take is from the interior, individual upper left quadrant (Figs. 2 and 3, S1). There can be no ‘view from nowhere’ – no truly objective stance. And yet, as we’ll see later in the paper, the implicit step taken by most scientists is that the interior experiences described by the upper left quadrant can emerge from the exterior behaviours of the upper right quadrant. i.e. that conscious inner life can emerge from inanimate matter. This is not science, it’s a hidden and unexamined metaphysics that is being increasingly recognised to be undermining scientific progress itself (McGilchrist 2021).

If unacknowledged, such a hidden metaphysics is also debilitating as it undermines our ability to relate to groundwater anymore in embodied ways; the ‘thingification’ of nature inherent in this worldview leads us to separate ourselves from having a relationship with ‘groundwater as nature’ and inherently leads us into a largely exploitative view of ‘groundwater as a resource’. The view of nature implicit in this perspective that separates human experience from the eternal environment, can undermine and undervalue more participatory and relational ways of experiencing, understanding and valuing groundwater. So what is being highlighted here is how important it is that we remember that such definitions of groundwater are abstracted concepts that arise as a social process of generating scientific knowledge; the paper aims to convince or remind you that groundwater is not something that exists independently of us experiencing it (Barad 2007), but rather that it is better thought of as a relational process.

The next section takes a step back from dearly held hydrogeological notions and framings of what groundwater is to consider ourselves in the sweep of history for, alongside modern groundwater, exist myriad perspectives regarding what groundwater might be. These are rarely considered in the hydrogeological community, though recent developments in socio-hydrogeology have been challenging ‘business as usual’ at least in regard to the legacy of colonialism, and exploring deeper and more open engagement with other worldviews and cultural paradigms (Bourke 2025).

To unpack how modern groundwater evolved, therefore, we need first to acknowledge any unexamined assumptions we hold, several of which are pointed out above. Then, let us in the next sections step back into deeper time and re-imagine an alternative narrative which is hopefully not only more true to the evidence, but also more useful for living well in the present.

### Major patterns in the historical evolution of human-groundwater thought-worlds

**Fig. 4:**
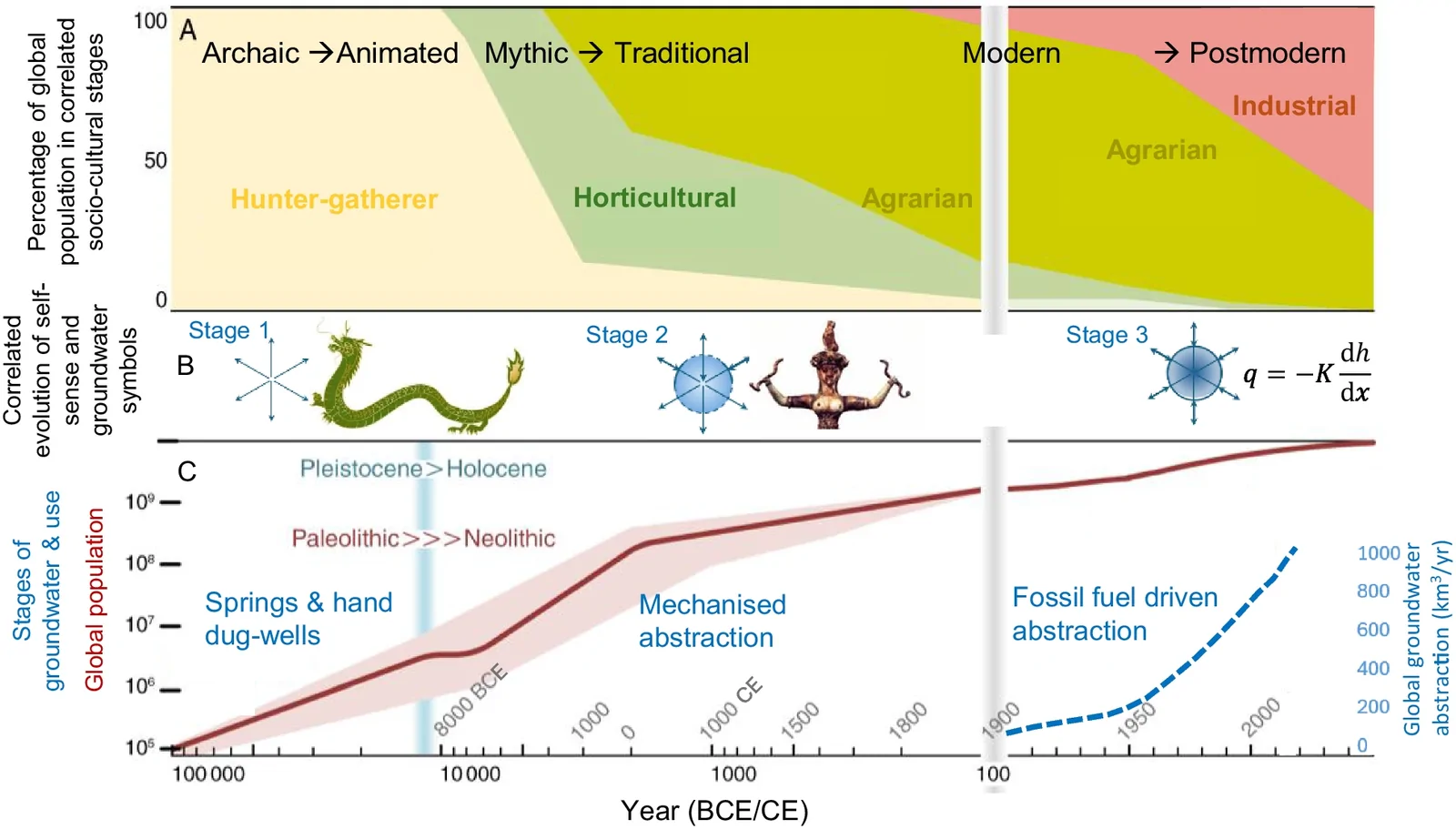
Historical correlations between stages in cultural, systemic, psychological and groundwater behaviour as identified in the quadrants of Fig. 3. Adapted with permission from (Ellis 2015). Groundwater withdrawal data from (Wada 2016)

In what follows, based on the combined integration of the scholarship outlined in the methods and ESM, a 3-stage overview for the evolution of modern groundwater is described, which parallels the evolution of modern human consciousness through stages called in this paper ‘unitive participation’, ‘participative duality’ and ‘separative duality’. The evolution of each quadrant is summarised in Fig. 3 and also expressed in overlapping timelines in Fig. 4. A tentative proposal for a metamodern turn for groundwater and a sensibility for what an emerging Stage 4 might look like as a ‘trinitarian re-imagination’ follows in Section Discussion.

#### Stage 1: Relationship with groundwater in unitive participation

We now have a growing body of evidence for hominin use of springs, possibly by australopithecines from around 4.5 Ma BP, but more confidently by the first emergence of the genus *Homo* around 2 Ma BP (Ashley et al. 2009). Our hominin ancestors in this stage are thought to have been developing beyond MHC stage 4 (Shah et al. 2020) common to many mammals, and perhaps up to ‘pre-operational’ levels of cognition (MHC stages 6–7) enabling sequential imitation and the ability to find relations among concepts allowing proto-language development. These aspects are speculative and highly debated but the precise timing and mechanisms are not key to the overall argument in this paper. Early *Homo* life was thought to be organised in small foraging bands and tribes which would have formed the primary cultural unit of ‘identity’, and there is evidence of developing cultural expression in increasingly sophisticated and beautiful stone tool crafts.

A key factor to have enabled development of increasingly sophisticated relationships with groundwater would likely have been through instinctive mimesis of animal behaviour such as elephants and co-extant great apes. Key here is the ability of hominins not just to mimic other beings, but also to sense, and mimetically enact the *intentions* being observed via the use of mirror neurons (Kaplan and Iacoboni 2006). Hence it is likely that our hominin ancestors also dug shallow hand-scooped wells in ephemeral streambeds, since they would have had the cognitive ability to learn from other mammals with a longer history of water-digging sense and skill such as elephants. Modern day chimpanzees use simple tools to dig for water in ephemeral stream beds adjacent to stagnant ponds (McGrew et al. 2013), and we can surmise that early hominins may have also developed this ability. This capacity for mimetic desire may also have driven the earliest vestiges of culture through tribal violence, re-cohesion and competition (Girard 2017, Burgis 2021).

The most archaic activity of the hominin mind in the representation of what WEIRD cultures now call the apparently external world can be conceived, e.g. following Barfield (1988), as a combination of (a) the relationship between “*that being represented*” (i.e. the external world), and the sense organs generating those sensations, and (b) a fabrication of those sensations, within the percipient’s mind, into (what we now call) ‘things’. This depends on a *phenomenally conscious* process i.e. there is “*something it is like*” to be experiencing those sensations (Nagel 1974). However, the process was not likely yet a *meta-conscious* one – i.e. hominins were not yet self-reflective and there was very little or no ability for ‘*thinking about’* (see the ESM).

Internal life was thus non-differentiated from nature, immersed in purely instinctive modes of being. With respect to the instinctive need to find and drink groundwater we might say that there would have not yet been an experience of ‘being thirsty’, but solely that of ‘being thirst’. Further there were not yet any abstracted concepts of objects, but rather just immersion in the flow of inner life (watery pun intended) that was assumed common and inherent to all phenomena arising in hominin awareness. At this stage early hominins had a relationship with groundwater that was intimate and immediate, although the ability to tell stories and mentally ‘time travel’ may have already been developing (Murray et al. 2017, 2019). Thus, this first and archaic stage is characterised by a relationship with groundwater that could be described as an instinctual and unitive participation.

#### Stage 2: Under-standing groundwater in participative duality

Following the emergence of the first anatomically modern humans (a few 100 ka BP) we eventually have the first possible evidence of deep supported hand dug wells (c. 100 ka BP, Knight (2022)), although this is a minimum date as the preservation potential of such structures is likely minimal depending on construction materials used. Art and language require greater cognitive complexity enabled by the increased brain size and structure of anatomically modern humans (AMH) and a blossoming of cultural sophistication eventually evidenced in cave art (~50 ka BP). Murray et al. (2017) hypothesise that modern humans developed an ‘explicit memory’ system from previously developed distinct representational systems that several ancestors had developed in the distant past. Explicit memory is defined by them as *“subjective perception of participating in events or knowing facts”*. This led, for the first time we know of in evolutionary history, to the ability for an individual mammal to develop a repository of conceptual knowledge.

The representation of self that is involved in the early development of explicit memory would have involved a kind of knowing that was occurring phenomenally as part of subjective awareness, but this still is not necessarily accompanied by meta-consciously ‘knowing *that* one knows’. The ability to conceive meta-consciously of the separation of self and other would have developed as cognitive complexity increased to ‘primary’ (MHC stage 8) levels. However, this emerging meta-conscious process of thinking would still have been carried out within a largely *participative* mode of being defined as a “*felt union with the inner origin of outward forms*” (Barfield 2014).

We can hypothesise then that early *Homo sapiens* had a relationship with water that was still intimate but less immediate, with an increasing ability to bring water into stories and planning, and the first vestiges of conceptual thinking about water was possible. The primary characteristics of this stage from an upper-left quadrant perspective are therefore (a) the evolving ability to phenomenologically participate in events leading to more nuanced and deeper/higher levels of a felt sense of self, and (b) the gradual development of the ability to re-represent oneself in mind meta-consciously. In essence, humans brought their own subjectivity into being by polarizing the world gradually into a duality (Barfield 2006).

The characteristic of this stage is one of humans learning to understand the behaviour of, and then control, groundwater through a mode of consciousness which was at once participative and dualistic with respect to the sense of self and other. Given the breadth of these developments, and their profound historical consequences, some provisional substages are defined for this stage below, though their boundaries are very porous and should be seen as a patchwork mosaic not a proposal for distinct time-space bound categories. As indicated in the methods, the primary focus in this paper is the three overarching stages, but it seems important to begin to explore possible substages here in addition, to at least provoke further scholarship.

*2a: Animated groundwater:* Understanding ancestral cultures that first developed tens of thousands of years ago in the upper Paleolithic c. 50 to 12 ka BP (before present) is challenging. Our only insights into the inner life of those communities can come from tentative and inferential based hypotheses drawn from studying the artwork and recorded oral traditions of now extinct, or a small number surviving, indigenous cultures which may or may not have direct links to our own cultural pathways. Strang (2023) beautifully catalogues abundant imagery and myths of water-beings from ‘place-based’ societies the world over, showing the ubiquity of water serpent beings associated with sacred places of groundwater emergence such as springs, oases and wetlands across all continents. These serpentine beings evoke the early sorts of relationship between humans with groundwaters that may have originally been experienced.

It is usually assumed that Paleolithic cultures shared strong similarities with surviving indigenous tribal cultures that are often described as ‘animist’. However, this term is much mis-used and normally misunderstood as being “*the imputation of life to inert objects*” (Ingold 2006) rather than a participative mode of being in the world (e.g. “*participation mystique*” (Lévy-Bruhl 1926), or “*original participation*” (Barfield 1988)). Animacy is “*the dynamic, transformative potential of the entire field of relations within which beings of all kinds… reciprocally bring each other into being*” (Ingold 2006). So, we might justifiably imagine our ancestors’ experience of groundwater to have been animated in a very real way, inhabiting a different type of perceptual and thought world altogether in comparison to our own, modern, world. Their serpentine water beings were consensus-realities in which their relationships with local groundwaters were lived out, powers with whom they related and negotiated, amongst the other natural beings that inhabited their every-day world. *They were not just projecting beliefs of a spirit world onto the same perceptual reality we inhabit today, but were living in a distinctly different perceptual mode*.

Despite their participative experience of the world, the new found ability for early *Homo sapiens* to treat objects of perception as if they existed independently of the perceiver through the habit/practice of a meta-conscious ‘self-other’/’subject-object’ conception was of obvious selective advantage. For example, this ability would have enabled improved strategies for navigating to water sources, designing tools for digging and stabilising hand-dug wells, for carrying groundwater and for developing strategies for remembering which sources were more reliable in different conditions and passing on such knowledge through story, song and visual imagery. Thus, we can hypothesise that this kind of thinking, as a mode of consciousness, became gradually more and more habitual and widespread for early humans given the selective advantage of traits including new ways of conceiving of, and thus successfully accessing, scarce groundwater drinking sources. This stage is historically characterised by increasingly complex/large tribal structures which would have formed the predominant foci for newly emerging individualised selves to have formed a cohesive sense of belonging and identity. Likely mostly working in a foraging mode of subsistence there was also the first appearance of larger villages, with increasing global dispersal and population though with likely still less than about 1 million humans on Earth (Figs. 3 and 4) when this stage dominated human culture.

*2b. Mythic groundwater*: If we are right to hypothesise that the earliest *Homo sapiens* participated in an animated world through the “*felt union with the inner origin of outward forms*” (Barfield 2014), the transition towards the mode of consciousness that characterises modernity must have involved the erosion of two experiential components – (1) that of perceived union with the rest of nature and (2) that of the perceived interiority of the rest of nature. The ways in which this happened is presumably as complex as the demonstrably creative experimentation of societal structures that characterised what we can piece together of human history (Graeber and Wengrow 2021). However, there is widespread evidence of a clear shift in ‘water beings’ taking on more human, and less serpentine, forms concurrent with the development of irrigated agriculture from around 12 ka BP (Strang 2023). Later, during one historic outworking of that which this study is calling the ‘mythic groundwater’ stage, by around 3000 BCE (Before the Common Era) the first stories also begin to appear whereby human heroes can exert their own power in the world and rebel against the nature gods. Such stories (e.g. the Babylonian Epic of Gilgamesh, the Indo-European pantheon gods of Greeks and Vikings) betray a parallel development in the inner world of humans, who can now more skilfully objectify and separate themselves from apparently external objects of perception, with increasing mastery and control over water in the outer world, through early water engineering technology. Increased cognitive abilities (first appearance of MHC stage 9, concrete) enabled innovations such as canals to move water from rivers or groundwater sources such as springs to irrigate areas for horticulture, and support increasing population densities as larger population centres and cities became established. Many of the great cities of the newly emerging states and empires were founded in areas dependent on groundwater and cultures living out this stage began to develop highly innovative ground water technologies as is well documented elsewhere (Fetter 2004a, b, Gleick 2023).

During this stage, cognitive complexity is sufficiently developed that the conception of phonetic alphabetic scripts becomes possible and, by 1500 BCE (Abram 1997) now there could be a singular notion of a ‘spring’ which could be thought about apart from all the place-specific springs that might be present in the actual local landscape. Despite this degree of conceptual abstraction developing, the proliferation of gods and goddesses that were associated with springs and other water ‘bodies’ betrays the continuity of some kind of participative reality in the human psyche. Humans perhaps symbolically named such beings not as objects in the way we might today, but rather as a set of interrelated phenomena which have an internal functioning just like their own interiority. Thus in this stage there was only a partial transition away from a purely participative mode of consciousness in the previous animated stage, towards a separation of nature into beings with their own non-human but nevertheless ‘enchanted’ form. Thus, we have the gods and goddesses of springs and wells which are expressed in many forms of European paganism and the spread of hydrolatry as “a single religious motif spanning the whole Eurasian continent and beyond” (Rattue 1995). Such water cults are still found in the present day in parts of Indo-European neo-pagan culture (Strang 2023), though the presence of such deities is arguably no longer a ‘real’ part of consensus reality in such communities owing to the shifts in consciousness discussed below.

*2c Traditional groundwater*: As cognitive capacity increases to MHC stage 10, abstract concepts and reasoning are deepened and the process of objectifying the apparently external world becomes increasingly habituated so that abstract thinking becomes part of culture and consensus reality. It then becomes both possible, and eventually natural, for the notions of god and goddess to also become more and more abstract and disconnected from particular places and then projected onto the cosmos at large, or even projected beyond nature to become entirely supernatural entities. Beginning around the 8^th^ to 5^th^ centuries BCE (sometimes known as the ‘axial age’ e.g. see (Jaspers 1953)), and culminating with the consolidation of the great religious traditions between that time and approximately 500 CE, we see the emergence of the monotheistic notion of God. In some sense this was the ultimate abstract concept as “God above all gods” (Freinacht 2017), but not in the sense of God as a separate all powerful being, supernatural creator or first cause - this is a recent, modern notion (Armstrong 2010). Rather the emerging monotheistic God was, variably across different cultures, seen as a symbol of indescribable transcendence, an absolute truth beyond everyday reality, but also one that was fully immanent either as nature (pantheism) or acting through nature (panentheism) (Culp 2023).

During the historical evidence for this stage Strang (2023) evidences a shift in water consciousness away from matriarchal traditions who venerated feminine water goddesses and beings, towards more patriarchal systems in which the feminine principle was embodied rather as loathsome watery demons. Despite the ordered ‘masculine’ dominance of the emerging monotheistic cultures of the Middle East, there remained a lingering fear of the great ‘feminine’ watery chaos (tehomophobia) deep within the hidden collective consciousness which played itself out in many interesting but often violent ways. For example, this shift in consciousness may have led, in my homeland of Wales, UK, to the Druid massacre of AD 60 symbolically putting to death the pagan world-serpent (Cunliffe 2010). This occurred at a time when the high status of women in Celtic Britain was widespread evidenced by pervasive matrilocality (Cassidy et al. 2025). More broadly in the UK and Europe, water worship began to be forbidden from at least the 5^th^ century CE onwards (Bord and Bord 1985). There was a widespread Christianisation of sacred pagan springs and wells to become ‘holy wells’ (Linton 2010, Misstear 2023) which were then used as a means of social control that persisted through the middle ages and only collapsed in the late 19^th^ Century (Rattue 1995). Eventually, there was a medieval period of ‘dragon slaying’ – perhaps a symbolic expression of the demonisation and repression of the serpentine feminine principle in some more politically co-opted expressions of the Abrahamic religions. Despite this, the motif of the watery underworld is still present in what is known as the ‘hydro-theological cycle’ whereby the entire water cycle can be seen to be metaphorically reflected in the life of Christ as expressed in the theology of these times (Linton 2010).

What appears to be happening in this stage, expressed historically for example in the European examples above, is a move towards more abstracted and meta-conscious modes of being, and a loss of appreciation of the deeper layers of transpersonal (i.e. the “collective unconscious” *sensu* Jung), participative modes of being in the world. This rise of what has been called ‘onlooker consciousness’ (Lehrs 1958) was characterised by some notable pockets of particularly increased prevalence of such left-brain dominant, analytical modes of thought (McGilchrist). In Europe this is perhaps best exemplified by the emergence of Greek schools of philosophy, and the aligned hypotheses that sprung up in antiquity about the behaviour of water – the first vestiges of what we now call the water cycle (Koutsoyiannis and Mamassis 2021). These early scientists applied logic, reason and analytical-thinking. However it would be a mistake to think that this was carried out within a perceptual mode of consciousness identical to that of those wearing the lenses of modernity. The philosophical process of ‘thinking about thinking’ was still likely done by early philosophers including Plato (*c*.400 BCE) while retaining a sense of original participation (Loftin and Leyf 2023). Hence the curious (to us) mixture we find in the texts from antiquity through to the early medieval period whereby logical observation and inference about the behaviour of nature is carried out within the lingering experience and/or assumption of the interior life of nature which governed their behaviour.

While there is a general move during Stage 2 from local water beings to serpentine deities to humanised or hybrid deities and finally ‘high gods’, there is also a mosaic of interactions between animated, magic and mythic cultures, as seen in the complexity of societal experiments (Graeber and Wengrow 2021) and the astonishing array of hybrid human-water beings (Strang 2023). More research is needed to integrate recent developments in scholarship on cultural evolution (Waring et al. 2015, Wilson 2020, Boyd and Richerson 2024) with more research on the socio-hydrogeological aspects which this paper has begun to identify.

#### Stage 3: Control and dominance over groundwater in separatist duality

*3a. Modern groundwater*: We now come to consider the mode of consciousness in which hydrogeological science as we know it was born, and within which the notion of groundwater itself is first articulated as an abstract substance/concept. From the participative unity of Stage 1, our ancestors moved through Stage 2 wherein some vestige of participation in nature survived until, eventually, in Stage 3 human beings experience themselves as atomised individuals, cut-off from the rest of nature inhabiting ‘onlooker consciousness’ (Lehrs 1958). Historically speaking, participation died very slowly between the Greek and Medieval periods – a difference in degree and not kind – until it was dealt a death blow in the scientific revolution (Barfield 1988). During this stage, important perceptual shifts also developed as the habituation of a different perceptual world gradually emerges from participation to onlooker consciousness. *Eventually humans came to believe that the abstract symbols and modelled quantities which they used to represent reality were actually reality itself*. As inheritors of this “*bifurcation of nature*” (Whitehead and Douchement 1920), our felt sense of separateness from nature has become automatic, as has the modern assumption that most of the rest of nature is inanimate i.e. has no interiority. Nature became a mechanical ‘clockwork’ universe, blindly following the laws of nature (Burtt 2014, McGilchrist 2021) and our attunement to the world became technological rather than poetic (Abram 1997, Harpur 2007).

Hydrogeology has developed within this modern cultural milieu during its emergence over the past 200 years or so. Following Linton (2010) we might say that “*modern [ground]water is the presumption that all [ground]waters can be and should be considered apart from their social and ecological relations and reduced to an abstract quantity*” ((Linton 2010)[my additions]). Modern groundwater is now commonly defined and related to as a resource, a ‘thing’ devoid of intrinsic meaning or value, separate from ourselves, which can be manipulated to our own ends. It is this kind of groundwater which myself, and I suspect many, if not most, other readers of this paper, are swimming in, but maybe blind to in one respect or another (Fig 1).

The predominant physicalist ontology of modernity has created, in groundwater, such a powerful conceptual representation that seemingly incredible benefits have accrued to society globally via our ability to understand and relate to groundwater in this mode. Training in MHC stage 11 (‘formal’) cognition became established within school education systems, and most university degrees are working at MHC stage 12 (‘systemic’). This combination of felt separation and cognitive training has underpinned the scientific endeavour by strengthening our capacity to conduct empirical investigations via inter-subjective verifiability and hypothesis testing (Ladyman 2012).

However, as also noted in the Introduction, the success of the adaptation of increasing mental ability to work with abstract notions of groundwater, has been paralleled by ever increasing physical abstraction of groundwater with widespread damaging consequences which don’t need to be repeated here (Gleeson et al. 2020). These problems sit within a wider problem of WEIRD (Henrich et al. 2010a, b) societies’ obsession with growth, coupled with its ability to invent technology to drive that growth without due consideration of the consequences. This is surely a *maladaptation* of the most serious kind now that we are over-reaching the boundaries of the planetary system on which we depend.

Most descriptions trace the pivotal culmination of the origins of the modern mind to a philosophical Cartesian dualism combined with an ever increasing systemization of rational thinking and empirical investigation of the natural world during the scientific revolution (c. 16^th^ century). However the historical trajectory leading up to this period which led to the paradigm shift at that time (*sensu* Kuhn (2012)) are critical to also understand. For example, Heinrich (2020) outlines how western Christendom’s reorganisation of kinship systems through the Middle Ages (c. 800–1200 CE through what he calls the “Church’s Marriage and Family Program”), induced critical social and psychological changes that enabled the rise of: mobility and increasing weakening of ties to ‘place’, individualism and analytical thinking, and impersonal pro-socialisation and marketisation.

The rise of the printing press in the 15^th^ century resulted in the spreading of phonetic alphabetic culture whereby “*the participatory proclivity of the senses was simply transferred from the depths of the surrounding life-world to the visible letters of the alphabet*” (Abram 1997). Around this time we see tell-tale changes in language, art and perceptions of time that betray the gradual shift in human consciousness. For example, the cyclical nature of time was replaced by a linear, abstract, notion of time enabled by an equally abstract notion of ‘space’ (or ‘extension’) as opposed to the particularity of *a* place (Abram 1997). A concurrent shift was occurring in the ability to abstract reality into more ‘representational’ forms of art during the Renaissance period. In the interior quadrants, western cultures have devalued participatory and perspectival modes of knowing in favour of procedural and propositional forms of knowledge (Vervaeke and Mastropietro 2021, Vervaeke 2024). This is mirrored by McGilchrists’s hypothesis of a gradual but persistently deepening neglect of the right hemisphere of the human brain: “*The left hemisphere is not in touch with reality but with its representation of reality*.” (McGilchrist 2012) and yet it has been given free-reign while sidelining the right hemisphere’s more holistic and connected view of reality itself. By the time of Copernicus, the stage was set for the paradigm shift that ensued, but this had at least a 1000 year build up – the Enlightenment thinkers were part of a long, cumulative process of cultural evolution that stretches back into late antiquity (Henrich 2020).

*3b. Postmodern groundwater*: Postmodernism represents a deconstruction of modernity via a critique of traditional and modern power relations, highly rationalistic thought, a suspicion of over-arching cultural and religious meta-narratives and truth claims, and an increasing embrace of global social and environmental concerns within a characteristically anti-hierarchical and pluralistic sensibility (Freinacht 2017). It is characterised by systemic thinking (MHC stage 12) and a suspicion of the modern notion of objectivity in truth. Its most obvious historical expressions have been since the 1950s, within art, philosophy, critical theory and the economics of post-war WEIRD societies. Postmodernity is still emerging as a candidate consensus worldview but a key development in postmodern thought is a focus on the fact that all phenomenological experience of reality is bound by social constructions. It is the realisation that every perception is only available to us via the use of representational symbols; reality is what we make of it.

In the postmodern context, Linton argues that “*Water is what we make of it*” (Linton 2010) and, by extension ‘postmodern groundwater’ could also be said to be what we make of it. A ‘post-modern turn’ for groundwater in recent groundwater research (of which this special issue of socio-hydrogeology is perhaps a part) is apparent in the increasing focus on global groundwater issues (Scanlon et al. 2023), and a pivot towards embedding groundwater within systems thinking (Gleeson et al. 2020, Huggins et al. 2023). There is more emphasis on social justice including the power dynamics of patriarchy and colonialism related to groundwater development (Zwarteveen et al. 2021, Bourke 2025). There is also increasing concern for the ‘more-than-human’ including moves, controversially, to extend the principles of human rights and personhood to water bodies (Clark et al. 2019). There has also been a widespread ‘goddess resurgence’ (Reid-Bowen 2016) which brings together post-modern sensibilities of the feminist movement and emerging *thealogies*. The associations of the goddess with springs and wells indicates a re-emergence of groundwater back into the collective psyche, albeit at a largely hidden level.

Postmodernity, as a potentially positive development in challenging the hidden biases, consumptive excesses and injustices of modernity, is nevertheless deeply mired in performative self-contradictions (Allen and Mendieta 2018). It undermines reality itself, becoming lost in a hypereality hall of mirrors whereby “*what passes for reality is a network of images and signs without an external referent*” (Aylesworth 2015). It takes even further modernity’s habit of mistaking the map for the territory, and concludes that there is actually no territory – only maps. Combined with extreme relativism, this leads to a disorienting stance by which its own methods undermine its own legitimacy, and tends to nihilism as all meaning is relativised. Part of the post-modern critique is also the undermining of the very notion of an autonomous self, outside of a social construction.

Despite being pushed back underground in the pre-modern and modern eras as noted above, groundwater beings are alive and well in postmodernity, especially in the visual and cinematic arts (e.g. a recent example being Disney’s Mandalorian who has a life changing encounter in the groundwater of the mines of Mandalore). While the explicit belief or direct experience of these beings is rare, they apparently still lurk in our hidden consciousness and express themselves in cultural forms – implicitly directing our behaviour from within. It is as if, just like a karst stream, our historical memory of serpentine groundwater beings disappears into the underworld, only to spring forth further downstream (Macfarlane 2019).

The deconstruction of the modern world that postmodernism provides, combined with multiple existential threats that the excesses of modernity has caused in the name of progress and growth leaves us in a place of meta-crisis, but also one of potential opportunity. Hydrogeology was born of modernity, but the world has moved on. It can’t cope with the complexity of the world territory we have created for ourselves in the 21^st^ century. We need to navigate it with a map that is more fit for purpose – at the very least for our survival, but hopefully in a world in which all can flourish, including the more-than-human reality we now are beginning to remember. The deconstruction of all our previous maps that postmodernism provides is only useful if those insights are then used to create better maps. To that possibility we now turn.

## Discussion

So far this paper has explored a proposed map of how WEIRD societies (Henrich et al. 2010a, b) have come to understand and relate to groundwater. Next, consideration is given to some of the emerging attractors in the future trajectory of groundwater-human relationships within the current global predicaments in which we find ourselves. What a metamodern turn might look like for groundwater is first considered before then proposing a particular metaphysical shift that may help us better work and ‘live with groundwater in mind’ as a possible imagination for what the metaphysics of Stage 4 for groundwater could look like.

### A metamodern turn for groundwater?

If the thrust of the argument so far has merit, at least in broad terms even if the details may be open to debate, it leaves us in an intriguing position. As stage-development theorists have indicated, if we transcend postmodernity by eschewing the rejection of metanarratives, but accept and include postmodern insights into the nature of such metanarratives, we are in a position to build a meta-metanarrative which may be a useful map for navigating our present global challenges but, unlike postmodernism, is also able to explain the existence of ourselves as the type of beings that are trying to explain it. This is the essence of the emerging metamodern social theory which is the central hermeneutical lens through which this paper is written (Vermeulen and Van den Akker 2010, Freinacht 2017, Andersen 2019, Björkman 2019, Freinacht 2019, Severan 2021, Storm 2021)

It accepts that, as expressed in history, the co-evolution of groundwater and human consciousness was no doubt a non-linear and complex process with many dead-ends and cultural forms that are now lost to us. However, this paper has argued that we can trace a pattern of transitions between three broad stages which tetra-evolved across the quadrants shown in Fig. 3. The process was one whereby, when sufficient numbers of individuals came to share a deep enough habituation of a particular state of consciousness (i.e. consciousness ‘*modes’* in the upper left quadrant), this became instantiated in a new normal, consensus reality (i.e. cultural ‘*codes’* in the lower left quadrant), iteratively reinforced by behavioural and systemic developments (i.e. interacting ‘*nodes’* of agents in right hand quadrants). Seeing the co-evolution of humans and groundwater in such a way is, potentially, the first move in a ‘metamodern turn’ for groundwater. Putting it in more ancient language of the Platonic triad mentioned earlier in which the quadrants can be shown to map, it is to ask ‘what is good, true, and beautiful about groundwater?’.

The metamodern paradigm acknowledges the postmodern insight that ‘all is narrative’, but attempts to move past this impasse by using the insights of modernism to decide which stories are better or worse with respect to a particular goal. “*To the modern mind, nature is the object… and culture is the subject…To the metamodern mind culture and nature are both part of the object, whereas the subject is the transpersonal development process itself*.” (Freinacht 2019). Hence, just as modern civilisation requires the governance of nature, metamodernism also requires the deliberate development of culture. As Graeber & Wengrow conclude “*We know, now, that we are in the presence of myths*” (Graeber and Wengrow 2021). So, given the complexities and potential existential threats posed by modernity’s technology, including our ability to pump so much groundwater that we have changed the Earth’s very axis, it is imperative that serious reflection is given firstly to ‘which myths are living us?’, and then ’what myths we want to live by?’. Clearly though, we must also grapple here with the paradox, like the ouroboros symbol of the serpent biting its own tail (Fig. 2), we can only evaluate what we want (i.e. what is ‘a good’ or ‘better’ narrative?) from within the parameters of the narrative in which we already live (Freinacht 2019).

What else might this mean for groundwater? A few aspects are outlined below which all require holding in tension multiple views, but encourage the community to bring the sensibility this paper has attempted to articulate so far to bear to go on to develop a deeper analysis:

#### Overcoming our blindness to interiors

We are already increasingly viewing groundwater systems as inherently embedded in socio-cultural and other Earth systems (Huggins et al. 2023), expressed through the behaviour of individual agents in the world, including humans and aquifers. But the metamodern turn will be one that addresses the imbalances in groundwater development from the perspective of *all* the quadrants (Fig. 2), in particular working towards a re-balancing of the left hand, interior, quadrants which have been almost entirely neglected in groundwater research. The challenges here are to (a) continue with the best of groundwater science with an increasingly deep focus on interactions with other systems while recognising that boundaries between systems are nominal, and perspectivally shaped by the position we ourselves as researchers take within a stage of development within a given quadrant, and (b) to acknowledge, understand and integrate insights from the interior forms of knowing with regard to psychological-cultural development pathways into our study of groundwater behaviours and systems. We may have finally articulated that we are now altering the water cycle wholesale, but at the same time we continue to assume it has always existed and was waiting there for us to *discover* it. We need to shift our perspective to rather realise that we *constructed* the water cycle, since it can only exist in relationship to the conscious minds who perceive its existence – we are part of it, not an outside agent who is altering it, and our interior life is an absolutely critical part of the water cycle we mustn’t continue to overlook. Embracing its evocative and sensual power through artistic expression may be one way of reconnecting to more embodied ways of knowing and relating to groundwater (Gleeson 2026).

#### Putting groundwaters back into ‘place’ and remembering deep and cyclical time

Here, the recent move to seek to understand and be guided by the insights from indigenous, place-based, culture is important, though fraught with difficulty and the trap of romanticisation. Understanding conflicts over groundwater within the context of differences in cultural stages, within and between national borders, is a powerful lens in which to reveal the metabolic friction of worldviews in contact. But, clearly the backdrop of colonialism looms large in such work (Bourke 2025), and a legitimate postmodern view on this that must be addressed head-on is that such a meta-modern perspective can become another type of post-colonial system of oppression. That said, two particular aspects of importance stand out. First, as WEIRD culture developed, an increasingly conceptual notion of groundwater was abstracted from actual landscapes:

“…*our organic attunement to the local earth is thwarted by our ever-increasing intercourse with our own signs*…” (Abram 1997).

Clearly there is power in such abstraction, and that is surely the key to any viable modelling exercise, but the recent habit of projecting large scale abstractions onto local problems is of concern (Cuthbert et al. 2024). A ‘de-universalising’ move to re-situate groundwaters back into their context of arising is needed (Dominguez Guzmán, et al. 2023). Second, once we appreciate the deeper time dimension of a phenomenon like groundwater, “*things come alive that seemed inert… and new responsibilities declare themselves*” (Macfarlane 2019). It is insightful that indigenous communities use multiple generations to determine their responsibilities, whereas it is rare for WEIRD societies to get beyond the next political cycle in real terms (known as the ‘hydro-illogical’ cycle (Sarah et al. 2019)), despite knowing that the timescales of most groundwater systems are intergenerational (Cuthbert et al. 2019). A challenge for metamodernity is also to hold together the tension between the more ancient view of cyclical time with the modern linear one.

#### Holding multiple definitions of groundwater as ‘both-neither’

A meta-modern turn will hold, in creative tension, multiple definitions of groundwater, and let them relate to each other within a dialectic as ‘both -neither’ rather than the post modern ‘neither nor’ (Vermeulen and Van den Akker 2010). For example, contrast the definitions found in the literature of groundwater as *“subsurface water that occurs beneath the water table in soils and geologic formations that are fully saturated”* (Freeze and Cherry 1979) with *“a hybrid materiality of grounds and waters… not as an objective fact…but as an actual being”* (Powis 2021). Or consider the visceral sense of groundwater as “*a dynamic architecture sucking and seeping, swelling and shrinking, absorbing and oozing”* (Ballestero 2019) alongside the idea of groundwater as a ‘hyperobject’ whereby *“consciousness of groundwater’s meanings enhances my understanding of this land and myself—my watery self, my underground self, my beneath the surface life*.” (Wardle 2019). Or consider a ‘work in progress’ definition of groundwater that the author is currently developing and working with:

“*Groundwater is a relational process of deep, hidden, fluid, connections between life and land across many generations and boundaries*”.

Scholarship on ‘what is water?’ more broadly (Linton 2010), in the context of natural- or chemical-kinds (Needham 2000, Weisberg 2006), or using the language or thinking *with*, as opposed to *about*, water (Chen et al. 2013) gives yet more perspectives for potentially exploring and applying to groundwater specifically.

#### Balancing the myth of the fall and the myth of progress

Two important persistent mythic motifs of ‘the fall’ and of ‘progress’ (Tarnas 2006, Wink 2017) form a helpful backdrop to the trajectory of the 3 stages outlined in the results section as follows (a) the *myth of progress*: whereby evolving conceptual and cognitive abilities enables humans to ‘master’ nature – this finds its most extreme form in techo-optimism (Alexander and Rutherford 2019) regarding the best way of dealing with the current climate and biodiversity crises of which groundwater is an embedded subsystem (b) *the myth of the fall*: whereby evolving meta-conscious awareness enables a felt sense of separation and alienation from the rest of Nature (Kastrup 2021a, b). This motif is central to the great spiritual traditions, (in particular their mystical expressions, and especially within the non-dual traditions (e.g. Wallis 2013, Hart 2024). Hence, while WEIRD consensus reality was moving in a direction of separation embracing the myth of progress, these traditions saw the problem of individual suffering as that of ‘separation’ in duality also being a primary cause of inter-relational and environmental suffering. Now we can see the extent to which that suffering is playing out at a global scale, both individually and corporately, and groundwater systems are entangled in this web of suffering. The non-dual insight into the nature of what it is to perceive and act as a human within a greater web of being is more needed now than ever given the global nature of our predicaments and the fact that there is ‘no planet B’ (Berners-Lee 2021). We are finally demonstrating ‘objectively’, using the tools of the right hand quadrants, what has been known for millennia via the tools of the left hand quadrants – that mindfulness based practices, especially when held within an ecology and community of practice, are profoundly effective in reducing suffering (Grossman et al. 2004, Hayes 2019). What is proposed here in this study is that we can hold in creative tension the power of both these myths. We can step back, become conscious of how these myths are ‘living us’ and integrate, knowingly, into a new story.

#### Embracing the relationality of groundwatering and its conscious evolution

Groundwater is an inherently relational concept of ‘water’ and ‘ground’. But more profoundly, groundwater only exists in so far as it is a relational process that we participate in – in as far as we conceive it, it is what we make it. It is a concept, and not something to be, necessarily, most usefully reified as an object, unless that is done so meta-consciously/explicitly. So, hydrogeologists are true ‘dis-coverers’ who bring ‘under-standing’ to the world as we descend and bring into the light those things that lie beneath (*quicquid subterra est*). As Barad puts it:

“*We are of the universe - there is no inside, no outside. There is only intra-acting from within and as part of the world in its becoming*.” so we must “*meet the universe halfway*” (Barad 2007).

We can meet groundwater half-way too, realising it is what we make it, but that it is also more than what we make of it. *We can thus participate consciously in the evolution of groundwater*. In the words of David Sloane Wilson:

“*No existing culture is adaptable enough to keep pace with the current changes so we need 'conscious evolution' to produce a new system of inheritance working at unprecedented spatial and temporal scale*” (Wilson 2020).

We need to add self-awareness to our evolutionary trajectory for improving Earth’s self-regulation (Lenton and Latour 2018).

One of the challenges we face in socio-hydrogeology in moving forwards in these ways, is a tension the author perceives between a modern cultural predominance within the hydrogeology community whereby most groundwater scientists work within a physicalist metaphysics, and a predominance of postmodern modes of thought within the social-science community. Moving genuinely into both ‘cross’ and ‘inter-disciplinarity’ must involve building strong and lasting bridges across this perspectival divide to pivot to a meta-modern view of groundwater which holds views not just from multiple quadrants, but also from multiple stages, at once or in oscillation. This kind of tension between worldviews is inevitable, and healthy debate and open dialogue in our communities on these issues would, surely, be a sign of progress.

### A metaphysical proposal for Stage 4 in which we re-source to live with groundwater ‘in mind’

It is uncertain whether or not something like metamodernity will continue to develop into a cultural stage of widespread significance or longevity. However, hopefully it has been demonstrated here that it represents a powerful and useful framework with which to think about global challenges and possible futures. Most such scholarly work on imagined futures focus on the right hand quadrants of Figs. 2 and 3 by re-framing the possible trajectories of human behaviours and systems into deterministic and (necessarily) highly uncertain storylines of, for example, the future of groundwater use in the context of climate, land use and demographic change. So, here in this last section of the paper, a completely different approach is taken to envisioning the future, inspired by the metamodern sensibility, with a starting point in the upper left quadrant of psychological development, that of interior human individual experience.

It is clear that our current Stage 3 philosophical and perceptual over-abstraction (left quadrants) is reflected in a material over-abstraction of groundwater (right quadrants), and that this is driven not just by a worthy need to support the water-food-energy system nexus, but by overconsumption through a culture that has lost a deeper sense of purpose and meaning (Barfield 2006, McGilchrist 2012, Mastropietro and Vervaeke 2020, Vervaeke and Mastropietro 2021). The metaphysics of philosophical materialism may be an important, and largely hidden, root of the current meta-crisis since it is not only a component of the meaning crisis but is also mutually entangled with the cultural materialism which is driving the climate and biodiversity crises (McGilchrist 2012). Furthermore, there are also deep empirical dissonances within the prevailing physicalist metaphysics of modernity (Smith 2022) which need to be met head on, rather than simply making room for competing and conflicting ontologies to co-exist.

The essence of a new cultural stage, seen within stage development theory as part of a growth hierarchy or holarchy (Koestler 1967, Wilber 1995), is that it will genuinely transcend and include the stages that went before it – it will bring new skill to living by transcending the problems caused by maladaptations of the previous stage, while creatively including and building upon on existing mental and physical structures. So, what might a Stage 4 for groundwater *feel* like for an individual, and what kind of world would they inhabit? This paper proposes Stage 1 to feel like a ‘unitive participation’ in groundwater i.e. “*a felt union with the inner origin of outward forms*” (Barfield 2014). Stage 2 then includes a complex set of transitions which begins the increasingly self-reflective separation of inner and outer experience in a ‘participative duality’. This process is seen in extreme form in Stage 3 when the interiority of, and union with, nature have both been almost entirely removed and the world is experienced as a ‘separative duality’. The conundrum we now face is this: our extraction and separation from, and the disenchantment of, nature is at the root of many of our current problems (‘the fall’), and yet this very separation has also enabled many incredible benefits to have accrued to society globally (‘progress’). So, can we imagine a way of gaining what we lost without losing what we gained?

Space precludes a detailed unpacking of such a vision here, which will be left for later publications. However, the reader is invited, as one way to articulate this, to imagine a possible Stage 4 as one of integrative ‘threeness’ or ‘trinity’, in which we can participate knowingly in our currently default mode of duality while not losing touch with a more primal mode of unity, and vice versa. Thus, the triad of ‘knower’, ‘known’ and ‘knowing’ are always present in the new mode, and the self is flexibly permeable to the relational field of interiority and exterior forms in which it flows. It is demonstrably possible for humans to experience a high degree of epistemic depth (i.e. a type of participatory experience of the world) without losing perceptual precision or the ability for a high degree of mental abstraction. Mystics have been embodying this for thousands of years, but something like this is only recently beginning to be rigorously described in the language of the right hand quadrants by scholars working at the intersection of neuroscience and spirituality (Laukkonen and Slagter 2021, Laukkonen and Chandaria 2025, 2024).

Every other culture that has ever existed (so far as we know) has directly recognised a deeper ‘source’ which animates reality and of which we are an apparent part within an integrated whole. Perhaps the closest we have come in the modern worldview to describe this source, is through the language of transpersonal psychology which recognises that our psyches are like outcrops of a deeper consciousness, part of which is an obfuscated personal consciousness (Kastrup 2017), but also a transpersonal obfuscated collective consciousness which is at the root of our shared world (Kastrup 2021a, b). It is as if we have come to view ourselves, and each other, as separate, superficial, bodies of surface water and forgotten that we are, in a more profound way, ephemeral ‘outcrops’ of a deeper body of groundwater which both sources and connects these streams across vast tracts of time and space. It seems long overdue that we shift our narrative away from groundwater, and nature more broadly, as being a resource, and rather reconnect to this source – to ‘re-source’ ourselves for the challenges we face as a global community.

Clearly the ouroboros rears its head (and tail) again here since an individual self can never be considered separate to the culture and physical systems in which it emerges, as its appearance in Fig. 2 is designed to remind us. But for now, you are invited to imagine a vision of what groundwater culture, behaviours and systems would look like within a collective of individuals holding this sense of self and what the language of such a shared groundwater imaginary might be. For those of us who share or aspire to develop something of such a sensibility, I hope we can explore this future together.

## Conclusions

This paper sets out to broaden existing narratives about what groundwater was, is and could be. By encouraging us to reflect on our own, potentially hidden, worldviews, it asks of us what becoming more conscious of these cultural lenses might mean for our future, collective, relationship with groundwater. It applies an evolutionary hermeneutic to explore how modern groundwater has come to be perceived, understood and defined within a particular, WEIRD (western, educated, industrialised, rich, democratic), cultural evolutionary pathway. It then proposes a new narrative for understanding the evolution of ‘modern groundwater’ in the context of evolution more broadly, including the evolution of consciousness. By considering the co-evolution of four aspects of human-groundwater development - behaviour, systems, psychology and culture – it proposes a metamodern framework which we may explore to find a new and wiser relationship with groundwater, and more effective responses to the predicaments of the Anthropocene.

This study draws on a weight of scholarship which indicates, since the birth of modernity, participatory ways of perceiving and knowing groundwater have largely given way to increasingly abstracted propositional modes of knowledge. For all the apparent progress this has brought us, in a very real way, we have also fallen into a maladaptive trap: our growing ability for mental abstraction has driven such massive physical abstraction (of groundwater and other ‘resources’) that we are in the midst of multiple global crises of (e.g. climate, biodiversity, meaning). We (WEIRD inheritors) progressively stripped the interiority first out of nature, and eventually out of ourselves - we have mistaken the map for the territory it describes by coming to believe that the abstract symbols and modelled quantities with which we represent and manipulate reality are actually reality itself. This impoverishment enabled the belief that exporting such a disenchanted worldview would transform the world for the better, first as colonists and now in perhaps even more far-reaching post-colonial power structures. The challenge proposed here for those of us who have inherited such a worldview is one of self-reflection, in an attempt to re-gain what we have lost (accepting the myth of the fall) while not losing what we have gained (accepting the myth of progress).

It has hopefully been shown that realising our need to better understand groundwater leads us to first better understand ourselves. To meet the global predicaments we face in the so-called meta-crisis, a meta-modern turn would pivot our focus away from sustainable development *of* groundwater towards ‘develop-mental sustainability’ *with* groundwater, which places our own inner development back in the frame as a critical component. Working hard as a community to continue to improve our hydrogeological understanding of groundwater systems to enable more sustainable groundwater management is still critically important. However, in the end this will surely not be enough: we don’t just need more modern hydrogeological thinking, but we need to actually start to think in new ways. As Einstein famously said “...*a new type of thinking is essential if mankind is to survive and move toward higher levels*.” (Einstein 1946). Ultimately it is the belief of the author that we need to finally transcend the physicalist metaphysics that is implicit in modernity, including most versions of its ‘post’ and ‘meta’ derivatives, and embrace a consciousness-first worldview which can not only strengthen the scientific endeavour, but restore meaning to the world and our place in it by ‘re-sourcing’ ourselves.

So, what then is groundwater? This paper is an attempt to make the case that groundwater is ultimately an idea, and it is therefore what we make of it as a relational and evolving process within an entangled web of behaviours, systems, psychologies and cultures. What we make of it depends on the lenses through which we perceive and think. As an invitation to the reader to co-create a new sensibility and relational framing in which to work with groundwater, and not to attempt a fixed definition, this form of words is offered:

*Groundwater is a relational process of deep, hidden, fluid, connections between life and land across many generations and boundaries*.

Here the focus has been on first how we might think, and therefore *be*. Deeper thinking about the directions in which we might then deliberately *behave* is of course an important future step, but these need to be based in new modes of thinking, being, perceiving and relating, and not just new ways of acting within the same paradigm. Asking ourselves individually and collectively what is ‘good, true and beautiful’ about groundwater may be a useful way forward for a mutually transformative, and truly inter-disciplinary, dialogue which could lead us in this direction. It is hoped that others will take these ideas on to make this provisional map fitter for purpose, without confusing it for the territory it is seeking to describe. This paper is an invitation to start by asking ourselves and each other (including our students, for those of us who teach), not just ‘*what* do you think?’, but ‘*how* do you think?’, about nature and about groundwater. That is surely the first step to working out how we can live purposefully with groundwater in mind, for the sake of future generations who will inherit our shared groundwater imaginary.

## Supplementary Information

Below is the link to the electronic supplementary material.

Supplementary file1 (PDF 390 KB)

## Data Availability

Not applicable.
