# Supplementary material for: What is groundwater?
Source: Hydrogeol J. 2026 Apr 1;34(6):1679–99. doi: 10.1007/s10040-026-03056-9 (PMC13562177; doi:10.1007/s10040-026-03056-9)
Supplement: Supplementary file 1 [file 10040_2026_3056_MOESM1_ESM.pdf]

Hydrogeology Journal: Electronic supplementary material

What is groundwater?

by Mark O. Cuthbert

School of Earth and Environmental Sciences, Cardiff University, Cardiff, UK

Email: cuthbertm2@cardiff.ac.uk

This supplementary material elaborates on the methods outlined in the main body of the paper and gives additional details of the primary literature used to develop them.

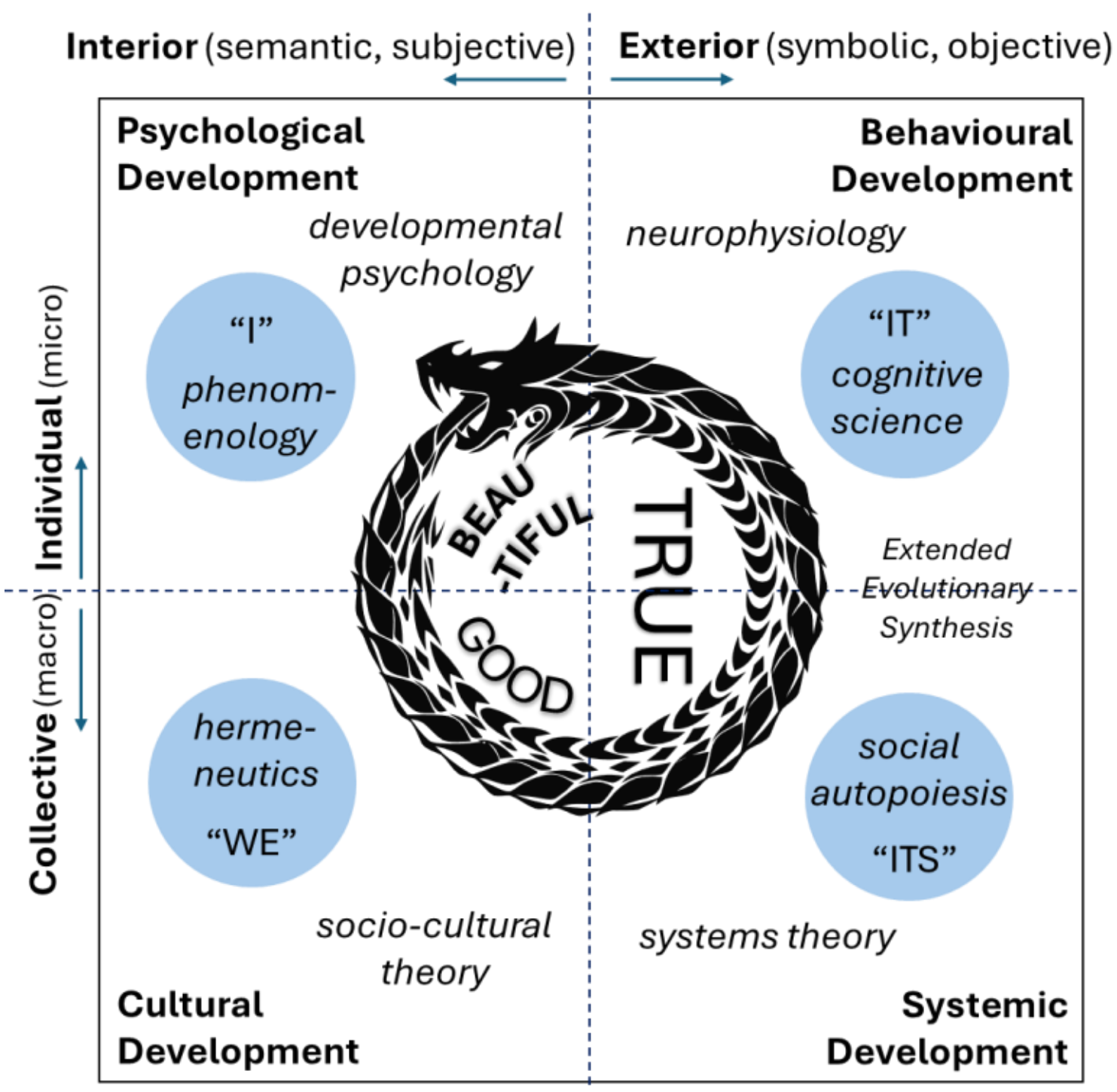

Figure S1. *Methodological details of the four fields of development (psychological, behavioural, cultural and systemic) defined by quadrants of interior/exterior and individual/collective development. The quadrants have been further divided internally according to 8 distinct categories of methodology employed in this study with respect to combinations of 1st or 3rd person perspectives with 1st or 3rd perspective approaches (i.e. italicized terms either inside or outside of the blue circles respectively) Esbjörn-Hargens and Zimmerman (2009). The correspondences to the Platonic triad of 'the true, the beautiful and the good' is also indicated, with an ouroborus to remind us that all four fields co-evolve and cannot ultimately be treated as separate from one another. The ouroboros image is licensed under https://creativecommons.org/publicdomain/zero/1.0/*

A quadrant map has been used to delineate four fields of societal development. Such quadrant maps (Figure S1, Figure 3) can be rich and powerful tools for almost any holistic analysis of the world including applications to sustainability science (Ives, Freeth et al. 2020, Shrivastava, Smith et al. 2020). Each quadrant can be viewed either as a different perspective or aspect of reality, or each representing different domains of enquiry, different types of truth claims, or specific methodological approaches (Wilber 1995).

Each quadrant has then been populated using a multi-level evolutionary framework which sees them interdependently co-evolve in time and space through developmental 'stages'. Stage theories for human and cultural development have the potential for misuse if not properly understood with openness, humility and sufficient nuance. Common confusions are to mistake developmental stages with particular historical time periods, failure to distinguish between natural growth hierarchies and dominator hierarchies, an assumption that later stages are somehow better in an objective or subjective sense to previous ones, and a confusion between features of the later stages which appear to share features of the earliest stages (the pre-trans fallacy). The reader is referred to chapter 8 of (Freinacht 2017), and references therein, as a useful primer. It is realised that, especially for those with a postmodern outlook, the suggestion that humans form growth hierarchies may be a problematic one. But this is something it seems critical to face, despite its potential for misunderstanding and misuse.

Relevant scholarship for each quadrant is now considered in turn, applying the methods outlined in Figure S1 in each case to build a rich and multi-faceted view of humans' co-evolution with groundwater.

Upper Right (UR): micro-exterior

In this 'behavioural' field of development I draw on work from the academic disciplines of evolutionary biology, neurophysiology and cognitive science. The following lines of thought and supporting literature are most central to my argument:

- Major anatomical developments in human evolution usefully delineate transitions from shared ancestors with Great Apes (c. 7 Ma BP) to early members of the genus *Homo* (*c*. 2-3 Ma BP), via various now extinct archaic human species to Anatomically Modern Humans (AMH) (*c*. 100-300 ka BP) (Stringer 2011, Dunbar 2014).
- The 'memory accretion model' is a state-or-the-art theory of the evolution of memory systems in humans in which exaption (i.e. repurposing) of previous brain functional developments, and various inherited representational systems involving memory, were eventually brought together for selective advantage in AMHs. In particular, the coming together of episodic and semantic memory systems in an 'explosive

generalisation' occurred across cognitive domains giving AMHs repositories of conceptual knowledge, rich autobiographical knowledge and cultural innovation (Murray, Wise et al. 2017, Murray, Wise et al. 2019).

- The Model of Hierarchical Complexity (MHC) is a well-tested framework which quantifies the cognitive complexity of behaviour such as verbal reasoning or other cognitive tasks. It has been shown to be valid across cultures and species and is based on the hierarchical nature of stage development (i.e. a given stage depends on the presence of the previous stage) (Commons, Trudeau et al. 1998). It is demonstrably robust when compared to other adult development stage theories (Tuladhar and Commons 2014).
- Multiple converging lines of scholarship from diverse fields evidence the lack of a truly 'separate self' and highlight the nominal division between the human 'self' and 'other'. This has been postulated biologically (Levin 2019), neurologically (Laukkonen and Chandaria 2024), psychologically (Jung 2014), phenomenologically (Laukkonen and Slagter 2021) and physically (Oliver 2020) at the level of the organism. The human organism appears to consist of nested processes which are physically measurable, which may or may not correlate to experience (upper left quadrant), and may or may not have access to verbal tools (McGilchrist 2021). A consistent picture also emerges from quantum information theory (Faggin 2024) and process philosophy (Whitehead 2010) that a powerful self-model may stem from conceiving the world as a superposition of nested processes and selves only being definable via 'agential cuts' made by observing processes within that universe (Barad 2007).
- There is a recent current in foundations of physics scholarship which explores the possibility that space-time itself is emergent from a deeper structure of reality (Seiberg 2006) that our evolution only enables us to perceive that which is evolutionarily useful for survival (Hoffman 2019) – we may then conceive of the upper right physical quadrant as a symbolic dashboard for navigating the world rather than giving access to the deepest structures of reality (Kastrup 2019).

Lower Right (LR): macro-exterior

In this 'systemic' field of development there is a rich seam of scholarship to draw from in development studies and 'Big History' which usefully lays out the terrain of major transitions in human development. The major lines of scholarship I draw on are:

- We must acknowledge that the realities of early human social and economic systems and their subsequent development were far more complex and interesting than most 'State of Nature' theorist generally acknowledge (Graeber and Wengrow 2021). Furthermore we must be aware of the possibility of

"retrospective coherence" whereby we may be tempted to "*perceive past events as more predictable, inevitable, or logically connected when viewed in hindsight than they were at the time of occurrence*" (Snowden and Rancati 2021).

- The theory of 'socio—cultural niche construction' (SNC) provides a coherent framework for understanding the evolution of systems of human production (Ellis 2015). This extends the Extended Evolutionary Synthesis, a framework for coupling cultural and ecological inheritance (Danchin, Charmantier et al. 2011, Danchin 2013), with the concepts of niche construction, ecological inheritance, human ultra sociality and cultural evolution.
- For our purposes here in giving a broad map of the patterns of the structural evolution of systems in which groundwater is now embedded in the modern worldview, I acknowledge the tension between theorists in this field, but will work within the paradigm that there is a long term trend towards *"the evolution of subsistence regimes capable of supporting ever-larger and denser human populations in increasingly unequal, hierarchical and complex societies by increasing land productivity over time through cooperative ecosystem engineering, increasing dependence on subsistence exchange over larger and larger distances, and by increasing use of non-human energy."* (Ellis 2015).

Lower Left (LL): macro-interior

The lower left quadrant is related to 'cultural' development,. However, the meaning of 'culture' is used differently between academic disciplines. For physical scientists there seems to be a tendency to assume definitions of culture defines culture as "*information transmitted across individuals through social learning*" (Danchin, Charmantier et al. 2011). This definition keeps culture in the right hand quadrants as exterior phenomena. Here, I use the term in a way more consistent with sociology which tends to define culture as shared, collective 'imaginaries' (Björkman 2019) – these are first person intersubjective entities that are manifest in shared symbol worlds, or 'codes'.

- The shared imaginaries determine our sense of what is real (Barfield 1988, Abram 1997, Harpur 2007, Kastrup 2016, Björkman 2019). Furthermore, sense data is interpreted according to our worldview unconsciously via our expectations and visually the world we experience meta-consciously may be effectively modelled as a 'controlled hallucination' (Koenderink 2011).
- The nascent metamodern movement in the arts, and philosophical literature proposes that there is a natural growth hierarchy within cultural evolution, just like in biological systems, whereby through the experimentation of variations in cultural forms, some survive or go extinct depending on their level of success

in being adapted to outcompeting other cultural forms (Wilber 1995, Freinacht 2017). What is initially a successful adaptation at an earlier stage of development can eventually become maladapted (Waring, Kline Ann et al. 2015, Wilson 2020, Boyd and Richerson 2024). Too much success at a given level of cultural development causes problems that then need to be overcome by a new cultural paradigm. The evolution of religious and mystical dispositions as well as formalised religions also appear to follow this pattern (Freinacht 2017, Dunbar 2022).

- Cultural evolution is tree-like, as in biology, with a massive variety of dead-end branches and the preservation, prospection and other biases of cultural information curation and exposition makes a statistically robust sampling of the cultural record impossible (analogous to the argument that "*sporadic sampling, not climatic forcing, drives observed early hominin diversity*" (Maxwell, Hopley et al. 2018)). Ecological niches of human groups during evolution map strongly onto the cultural stages outlined in the metamodern literature (Waring, Kline Ann et al. 2015) giving some indication that the patterns that seem present in world history are not just a product of retrospective coherence . Just as with biological evolution we should not be surprised to observe convergent evolution whereby similar forms of culture evolve in multiple locations at the same or different times (Boyd and Richerson 2024).
- The evolution of the human relationship with water is revealed in the evolution of distinct symbology throughout different stages of cultural development. This is most strikingly articulated by the fact that, despite the diversity of water beings across cultures, there are distinct patterns that co-emerge with certain socio-economic structures – for example in the advent of irrigated agriculture (Strang 2023).
- The cultural development of 'modern water', and its culmination in 'global water' described by Linton (Linton 2010) is directly applicable to the parallel development of 'modern groundwater' articulated more specifically in this paper. Modern water is *"the presumption that any or all waters are to be considered apart from their social and ecological relations and reduced to an abstract quantity"*. The development of modern water is seen as a gradual disenchantment of place-based 'waters' as inherently socially embedded processes, to the conceptual notion of 'water' as exemplified in 'the water cycle'.

Upper Left (UL): micro-interior

The upper left quadrant relates to individual psychological development (3rd person perspective) and phenomenology (1st person perspective). Since this quadrant is the basis of all epistemology, and the perceived sense of self is at the root of much human behaviour, it is incredible that it has not received more attention in the field of environmental sustainability. The following lines of scholarship are most central to my argument:

- Key to working in this quadrant is the term ‘consciousness’ which has a vast range of definitions (Kuhn 2024) and, as such, is particularly prone to misunderstanding so needs defining clearly from the outset. In this paper I use the term in line with the detailed analytical arguments of (Kastrup 2017) as follows: “*mental activity is conscious if, and only if, there is something—anything—it is like to have such mental activity in and of itself (Nagel 1974, Chalmers 2003)*”.
- The paper draws on the idea that there has been, and is ongoing, an evolution in the modes of consciousness experienced by the human psyche. Again consistent with the definitions used by (Kastrup 2019), I distinguish purely *phenomenally conscious* processes i.e. there is “something it is like” to be having experiences, from *meta-conscious* processes which involve self-reflection, or ‘thinking about’.
- The philological work of Owen Barfield shows that, in language, we find the fossilised evidence of consciousness evolution we need to interpret the past few thousand years of oral and written history (Barfield 1973, Barfield 1988). In essence Barfield argues that “*the evolution of nature is correlative to the evolution of consciousness; and, secondly, that the evolution of consciousness hitherto can best be understood as a more or less continuous progress from a vague but immediate awareness of the ‘meaning’ of phenomena towards an increasing preoccupation with the phenomena themselves*” (Barfield 1988).
- The evolution of the ‘self’ is a key line of development within this quadrant pertinent to the argument of the paper. Documented across many cultures over thousands of years, is the notion that there is available within the human psyche, with great reliably and reproducibility via the application of certain well tested mystical/contemplative practices, several distinct ‘modes’ of consciousness, which may persist as temporary experiences or become established as a permanent ‘stage’ of consciousness (James 1982, Wallis 2017, Ingram 2018). Key to this paper is the shift that can occur between perceiving the world in duality, into what is often termed ‘non-dual awareness’. It is proposed in this paper, and is a matter of direct experience for the author, that what is being pointed to as the non-dual state can transcend and include the unitive participation and separatist duality of the earlier stages that have also been described here.
- There is a growing neuroscience literature which is now mapping the neuro-correlates of such states of conscious experience via, for example, brain-imaging, and providing theoretical models of meta-conscious processes describing the relationships between these inner (UL quadrant) and outer (UR quadrant) observations (Laukkonen and Slagter 2021, Laukkonen and Chandaria 2024).

References

Abram, D. (1997). The spell of the sensuous: Perception and language in a more-than-human world, Vintage.

Barad, K. (2007). Meeting the universe halfway. Duke University Press.

Barfield, O. (1973). Poetic diction: A study in meaning, Wesleyan University Press.

Barfield, O. (1988). Saving the appearances: A study in idolatry, Wesleyan University Press.

Björkman, T. (2019). The world we create: From God to market, Perspectiva Press.

Boyd, R. and P. J. Richerson (2024). "Cultural evolution: Where we have been and where we are going (maybe)." Proceedings of the National Academy of Sciences **121**(48): e2322879121.

Chalmers, D. J. (2003). "Consciousness and its place in nature." The Blackwell guide to philosophy of mind: 102-142.

Commons, M. L., et al. (1998). "Hierarchical complexity of tasks shows the existence of developmental stages." Developmental Review **18**(3): 237-278.

Danchin, É. (2013). "Avatars of information: towards an inclusive evolutionary synthesis." Trends in ecology & evolution **28**(6): 351-358.

Danchin, É., et al. (2011). "Beyond DNA: integrating inclusive inheritance into an extended theory of evolution." Nature reviews genetics **12**(7): 475-486.

Dunbar, R. (2014). Human evolution: A pelican introduction, Penguin UK.

Dunbar, R. (2022). How religion evolved: And why it endures, Oxford University Press.

Ellis, E. C. (2015). "Ecology in an anthropogenic biosphere." Ecological Monographs **85**(3): 287-331.

Esbjörn-Hargens, S. and M. E. Zimmerman (2009). Integral ecology: Uniting multiple perspectives on the natural world, Shambhala Publications.

Faggin, F. (2024). Irreducible: Consciousness, life, computers, and human nature, John Hunt Publishing.

Freinacht, H. (2017). The listening society, Metamoderna.

Graeber, D. and D. Wengrow (2021). The dawn of everything: A new history of humanity, Penguin UK.

Harpur, P. (2007). The philosophers' secret fire: A history of the imagination, Blue Angel Gallery.

Hoffman, D. (2019). The case against reality: Why evolution hid the truth from our eyes, WW Norton & Company.

Ingram, D. (2018). Mastering the core teachings of the buddha: An unusually hardcore dharma book-revised and expanded edition, Red Wheel/Weiser.

Ives, C. D., et al. (2020). "Inside-out sustainability: The neglect of inner worlds." Ambio **49**: 208-217.

James, W. (1982). "The varieties of religious experience (ME Marty, Ed.)." New York: Viking Penguin Inc.

Jung, C. G. (2014). The structure and dynamics of the psyche, Routledge.

Kastrup, B. (2016). More Than Allegory: On religious myth, truth and belief, John Hunt Publishing.

Kastrup, B. (2017). "There is an 'unconscious,'but it may well be conscious." Europe's Journal of Psychology **13**(3): 559.

Kastrup, B. (2019). The idea of the world: a multi-disciplinary argument for the mental nature of reality, John Hunt Publishing.

Koenderink, J. J. (2011). "Vision and information." In 'Perception Beyond Inference' MIT Press.

Kuhn, R. L. (2024). "A landscape of consciousness: Toward a taxonomy of explanations and implications." Progress in Biophysics and Molecular Biology **190**: 28-169.

Laukkonen, R. E. and S. Chandaria (2024). "A beautiful loop: An active inference theory of consciousness." PsyArXiv. Retrieved from osf. io/preprints/psyarxiv/daf5n doi **10**.

Laukkonen, R. E. and H. A. Slagter (2021). "From many to (n) one: Meditation and the plasticity of the predictive mind." Neuroscience & Biobehavioral Reviews **128**: 199-217.

Levin, M. (2019). "The computational boundary of a "self": developmental bioelectricity drives multicellularity and scale-free cognition." Frontiers in psychology **10**: 2688.

Linton, J. (2010). What is water?: The history of a modern abstraction, UBC press.

Maxwell, S. J., et al. (2018). "Sporadic sampling, not climatic forcing, drives observed early hominin diversity." Proceedings of the National Academy of Sciences **115**(19): 4891-4896.

McGilchrist, I. (2021). The matter with things: Our brains, our delusions, and the unmaking of the world, Perspectiva Press.

Murray, E. A., et al. (2017). the Evolution of Memory Systems: Ancestors, anatomy, and adaptations, Oxford University Press.

Murray, E. A., et al. (2019). The Evolutionary road to human memory, Oxford University Press, USA.

Nagel, T. (1974). "The Philosophical Review." What is it Like to Be a Bat: 435-450.

Oliver, T. (2020). The self delusion: The surprising science of our connection to each other and the natural world, Hachette UK.

Seiberg, N. (2006). "Emergent spacetime." arXiv preprint hep-th/0601234.

Shrivastava, P., et al. (2020). "Transforming sustainability science to generate positive social and environmental change globally." One Earth **2**(4): 329-340.

Snowden, D. and A. Rancati (2021). Managing complexity (and chaos) in times of crisis. A field guide for decision makers inspired by the Cynefin framework, Publications Office of the European Union.

Strang, V. (2023). Water beings: from nature worship to the environmental crisis, Reaktion Books.

Stringer, C. (2011). The origin of our species, Penguin UK.

Tuladhar, C. T. and M. L. Commons (2014). "Correspondence between some life-span, stage theory developmental sequences of stages and levels." Behavioral Development Bulletin **19**(3): 24.

Wallis, C. D. (2017). The Recognition Sutras: Illuminating a 1,000-Year-Old Spiritual Masterpiece, Mattamayura Press.

Waring, T. M., et al. (2015). "A multilevel evolutionary framework for sustainability analysis." Ecology and Society **20**(2).

Whitehead, A. N. (2010). Process and reality, Simon and Schuster.

Wilber, K. (1995). Sex, ecology, spirituality: The spirit of evolution, Shambhala Publications.

Wilson, D. S. (2020). This view of life: Completing the Darwinian revolution, Vintage.
